# NLRP6 potentiates PI3K/AKT signalling by promoting autophagic degradation of p85α to drive tumorigenesis

**DOI:** 10.1038/s41467-023-41739-z

**Published:** 2023-09-28

**Authors:** Feng Zhi, Bowen Li, Chuanxia Zhang, Fan Xia, Rong Wang, Weihong Xie, Sihui Cai, Dawei Zhang, Ren Kong, Yiqiao Hu, Yilin Yang, Ya Peng, Jun Cui

**Affiliations:** 1 Department of Neurosurgery, Third Affiliated Hospital of Soochow University, Changzhou, Jiangsu China; 2 Guangdong Province Key Laboratory of Pharmaceutical Functional Genes, MOE Key Laboratory of Gene Function and Regulation, State Key Laboratory of Biocontrol, School of Life Sciences, Sun Yat-sen University, Guangzhou, China; 3 Medical Research Institute, Guangdong Provincial People’s Hospital, Guangdong Academy of Medical Sciences, Southern Medical University, Guangzhou, Guangdong China; 4 Institute of Bioinformatics and Medical Engineering, School of Electrical and Information Engineering, Jiangsu University of Technology, Changzhou, Jiangsu China; 5 State Key Laboratory of Pharmaceutical Biotechnology, Medical School and School of Life Sciences, Nanjing University, Nanjing, Jiangsu China

**Keywords:** Autophagy, CNS cancer, NOD-like receptors, Ubiquitylation, Oncogene proteins

## Abstract

The PI3K/AKT pathway plays an essential role in tumour development. NOD-like receptors (NLRs) regulate innate immunity and are implicated in cancer, but whether they are involved in PI3K/AKT pathway regulation is poorly understood. Here, we report that NLRP6 potentiates the PI3K/AKT pathway by binding and destabilizing p85α, the regulatory subunit of PI3K. Mechanistically, NLRP6 recruits the E3 ligase RBX1 to p85α and ubiquitinates lysine 256 on p85α, which is recognized by the autophagy cargo receptor OPTN, causing selective autophagic degradation of p85α and subsequent activation of the PI3K/AKT pathway by reducing PTEN stability. We further show that loss of NLRP6 suppresses cell proliferation, colony formation, cell migration, and tumour growth in glioblastoma cells in vitro and in vivo. Disruption of the NLRP6/p85α interaction using the Pep9 peptide inhibits the PI3K/AKT pathway and generates potent antitumour effects. Collectively, our results suggest that NLRP6 promotes p85α degradation via selective autophagy to drive tumorigenesis, and the interaction between NLRP6 and p85α can be a promising therapeutic target for tumour treatment.

## Abstract (web summary)

The crosstalk between innate immunity and autophagy plays a critical role in cancer. Here, the authors report that an immune receptor NLRP6 potentiates the PI3K/AKT pathway by selective degradation of p85α. The NLRP6-p85α interaction offers a potential therapeutic target for tumor treatment.

## Introduction

Class I phosphoinositide 3-kinase (PI3K) plays a central role in tumour development^1^. PI3K phosphorylates phosphatidylinositol-4,5-bisphosphate (PIP2) to generate phosphatidylinositol-3,4,5-trisphosphate (PIP3) followed by the recruitment of the serine and threonine kinase AKT^2^. As a frequent hallmark of cancer, PI3K activity is aberrantly activated by upstream factors, loss or inactivation of the tumour suppressor phosphatase and tensin homologue (PTEN), or genetic alteration or protein-protein interaction of PI3K subunits^3^. PTEN is the main negative regulator of the PI3K/AKT pathway, which regulates multiple cellular processes by dephosphorylating PIP3 to PIP2^4^. Under normal physiological conditions, the activity of the PTEN-PI3K axis is tightly controlled and maintained, while in cancer cells PTEN is lost or inactivated, and PI3K/AKT is hyperactivated^5^. Thus, PI3K/AKT pathway inhibition or PTEN upregulation may provide a new avenue for tumour prevention and treatment.

The innate immune system is the first line of host defence against pathogens to prevent infection and maintain homoeostasis through pathogen-associated molecular pattern (PAMP) recognition via pattern recognition receptors (PRRs), including C-type lectin receptors (CLRs), RIG-I-like receptors (RLRs), Toll-like receptors (TLRs), and NOD-like receptors (NLRs)^6^. Among the PRRs, NLRs are a specialized subset of intracellular proteins that recognize various ligands from microbial pathogens, host cells, and environmental sources in the innate immune response^7^. Several NLRs, such as NOD1, NOD2, and NLRP3, have been extensively studied for their roles in activating innate immune signalling. NOD1 and NOD2 can activate NF-κB signalling once they encounter their relevant PAMPs^8^. Several other NLRs, including NLRP3, NLRP6, NLRP1, and NLRC4, can form multimeric protein complexes known as inflammasomes, which mediate the maturation and secretion of the proinflammatory cytokines IL-1β and IL-18 as well as pyroptosis^9–11^. In addition, other groups of NLRs, including NLRX1^12^, NLRP4^13^, and NLRP11^14^, function as negative regulators to maintain the homoeostasis of innate immune signalling. However, no evidence has been shown that NLRs can directly interact with subunits of PI3K to control the PI3K/AKT pathway in glioma.

In this study, we identify NLRP6 as a dominant positive regulator of the PI3K/AKT pathway. NLRP6 potentiates the PI3K/AKT pathway through its direct interaction with p85α, the regulatory subunit of PI3K. Mechanistically, NLRP6 recruits the E3 ligase RBX1 to promote the ubiquitination of p85α at lysine 256 (K256), which is recognized by the cargo receptor OPTN and undergoes selective autophagic degradation. NLRP6-RBX1 represents a distinct E3 ligase complex that is different from the conventional Cullin-RBX1 ubiquitin E3 ligase complex. Moreover, NLRP6 expression is negatively correlated with p85α and PTEN in human glioblastoma. Disruption of the NLRP6/p85α interaction by the Pep9 peptide derived through the p85α secondary structure suppresses the PI3K/AKT pathway and inhibits tumour growth in vitro and in vivo. Taken together, our findings provide insight into the intricate regulation of the PI3K/AKT pathway and provide a promising therapeutic strategy for cancer treatment.

## Results

### NLRP6 potentiates PI3K/AKT pathway via p85α

**Fig. 1:**
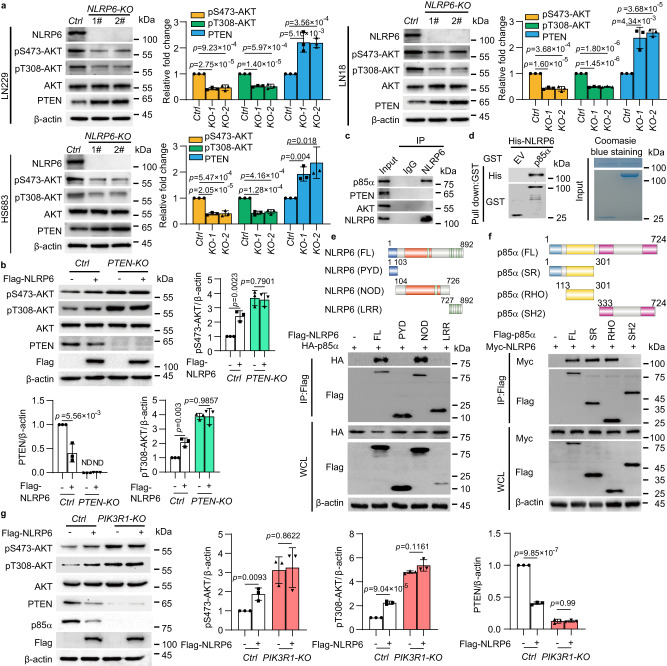
NLRP6 potentiates PI3K/AKT pathway via p85α. **a** Immunoblot analysis of cell lysates from control (*Ctrl*) or *NLRP6* knockout (KO) LN229, LN18, and HS683 cells and probing with indicated antibodies (left). Quantification of indicated protein levels (right). *KO-1*, *NLRP6-KO* 1#, *KO-2*, *NLRP6-KO* 2#. **b** Immunoblot analysis of cell lysates of *Ctrl* or *PTEN* KO LN229 cells and probing with indicated antibodies (left). Quantification of indicated protein levels (right). ND, not detected. **c** Coimmunoprecipitation (Co-IP) analysis of the interaction between endogenous NLRP6 and endogenous p85α, PTEN, and AKT in LN229 cell lysates. **d** Co-IP of purified His-NLRP6 with GST-p85α. EV, empty vector. **e** The domain organization map of NLRP6 (top) and Co-IP analysis to map the interaction between p85α and different NLRP6 domains in HEK293T cells (bottom). WCL, whole cell lysates. **f** The domain organization map of p85α (top) and Co-IP analysis to map the interaction between NLRP6 and different p85α domains in HEK293T cells (bottom). **g** Immunoblot analysis of cell lysates of *Ctrl* or *PIK3R1* KO LN229 cells and probing with indicated antibodies (left). Quantification of indicated protein levels (right). In **a**, **b**, and **g**, all error bars, mean values ± SD, *p*-values were determined by unpaired two-tailed Student’s *t* test of *n* = 3 independent biological experiments. Data are representative of three independent experiments with similar results (**c**, **d**), or two independent experiments (**e**, **f**). Source data are provided as a Source Data file.

To investigate whether NLRs could regulate PI3K/AKT pathway, the FOXO luciferase reporter assay was performed to detect the transcriptional activity of FOXO, which is one of the best characterized targets of PI3K/AKT pathway. Two independent oligonucleotides targeting each of 22 human NLRs were mixed and transfected into LN229 cells. The nontargeting oligonucleotides served as a negative control. The knockdown efficiency targeting each NLR was confirmed by qRT-PCR (Supplementary Fig. 1a). Among these NLRs, *NLRP6* knockdown showed most significant increase of FOXO transcription activity in LN229 cells (Supplementary Fig. 1b). As PTEN is the main negative regulator of PI3K/AKT pathway and the PTEN-PI3K axis plays important roles in a variety of human diseases^4^, we wondered whether the effects of these NLRs on PI3K/AKT activity were through the regulation of PTEN. We found that the protein abundance of PTEN was markedly increased by *NLRP6* knockdown compared with other NLRs (Supplementary Fig. 1c), while *PTEN* mRNA expression was not obviously influenced by *NLRP6* knockdown (Supplementary Fig. 1d). We next used cycloheximide (CHX) to block new protein synthesis and monitored the protein degradation rate of endogenous PTEN in a time-course experiment. *NLRP6* deficiency could prolong the half-life of PTEN protein (Supplementary Fig. 1e). These findings suggested that NLRP6 may activate PI3K/AKT pathway by promoting PTEN protein degradation. To confirm this finding, *NLRP6* was knocked out using CRISPR/Cas9 technology in LN229, LN18, and HS683 cells^15^. These three cell lines have wild type (WT) *PTEN* and are commonly used in glioma research^16–18^. As genetic loss or mutations of PTEN may generate dysfunctional PTEN leading to aberrant activated PI3K/AKT pathway^4^, *PTEN* WT cell lines were chosen in our research. p-AKT at Ser473 and Thr308 was significantly decreased while PTEN protein expression was significantly increased when *NLRP6* was knocked out in all three cell lines (Fig. 1a). The AKT pathway phosphorylation array was also used to assess the effect of NLRP6 on PI3K/AKT pathway by detecting the relative phosphorylation levels of 18 AKT pathway proteins. *NLRP6* deficiency significantly reduced AKT phosphorylation (pS473-AKT) and blocked the activity of its related signalling molecules p27 and BAD in both LN229 and LN18 cells (Supplementary Fig. 1f). MK-2206 is a highly potent and selective allosteric AKT inhibitor^19^. We found that NLRP6 overexpression could not activate PI3K/AKT pathway anymore when MK-2206 was introduced (Supplementary Fig. 2a). As PTEN protein stability was negatively controlled by NLRP6, we asked whether the effect of NLRP6 on PI3K/AKT pathway was solely dependent on PTEN. We found that NLRP6 could no longer enhance the level of p-AKTs in *PTEN* knockout (KO) cells, indicating the critical role of PTEN in NLRP6-mediated PI3K/AKT activation (Fig. 1b). Moreover, NLRP6 could not activate PI3K/AKT pathway in U251 cells, a *PTEN*-deficient cell line^20^ (Supplementary Fig. 2b). Taken together, these results suggested that NLRP6 could activate PI3K/AKT pathway and promotes the protein degradation of PTEN.

As NLRP6 activated PI3K/AKT pathway and promoted PTEN protein degradation, we wondered whether this phenomenon was through the direct interaction between NLRP6 with AKT or PTEN. Surprisingly, endogenous coimmunoprecipitation (Co-IP) assay revealed that NLRP6 did not bind with AKT or PTEN, indicating that other regulators were involved in NLRP6-mediated PI3K/AKT pathway activation (Fig. 1c). To identify NLRP6-associated proteins in living cells, we applied an APEX2-based labelling method combined with mass spectrometry (Supplementary Fig. 2c) and identified p85α (encoded by *PIK3R1*) as an NLRP6-interacting protein. It has been reported that p85α enhances the stability and activity of PTEN by its direct association with PTEN^21,22^. We also confirmed that p85α overexpression significantly enhanced the protein abundance of PTEN, leading to diminished PI3K/AKT pathway activation in LN229 cells, while this phenomenon was not observed in PTEN deficient U251 cells (Supplementary Fig. 2d). In contrast, p85α deficiency destabilized PTEN and promoted AKT phosphorylation in LN229 cells but not in U251 cells (Supplementary Fig. 2e). These findings were consistent with previous reports^21,22^. Furthermore, we found endogenous NLRP6 was coimmunoprecipitated with endogenous p85α but not with PTEN or AKT (Fig. 1c and Supplementary Fig. 2f). Moreover, purified GST-p85α directly binds with His-NLRP6 in vitro (Fig. 1d). To map the binding domains of NLRP6 on p85α, several Flag-tagged NLRP6 deletion constructs were cotransfected with HA-p85α. The NOD domain on NLRP6 was essential for its interaction with p85α (Fig. 1e). In parallel, the RHO domain on p85α was essential for its interaction with NLRP6 (Fig. 1f). Furthermore, ectopic expression of NLRP6 in p85α-depleted cells could no longer promote AKT phosphorylation or degrade PTEN, suggesting that PI3K/AKT pathway activation and PTEN degradation mediated by NLRP6 were dependent on p85α (Fig. 1g). Taken together, these results suggested that NLRP6 activated PI3K/AKT pathway and decreased PTEN protein stability via p85α.

### NLRP6 promotes the autophagic degradation of p85α

**Fig. 2:**
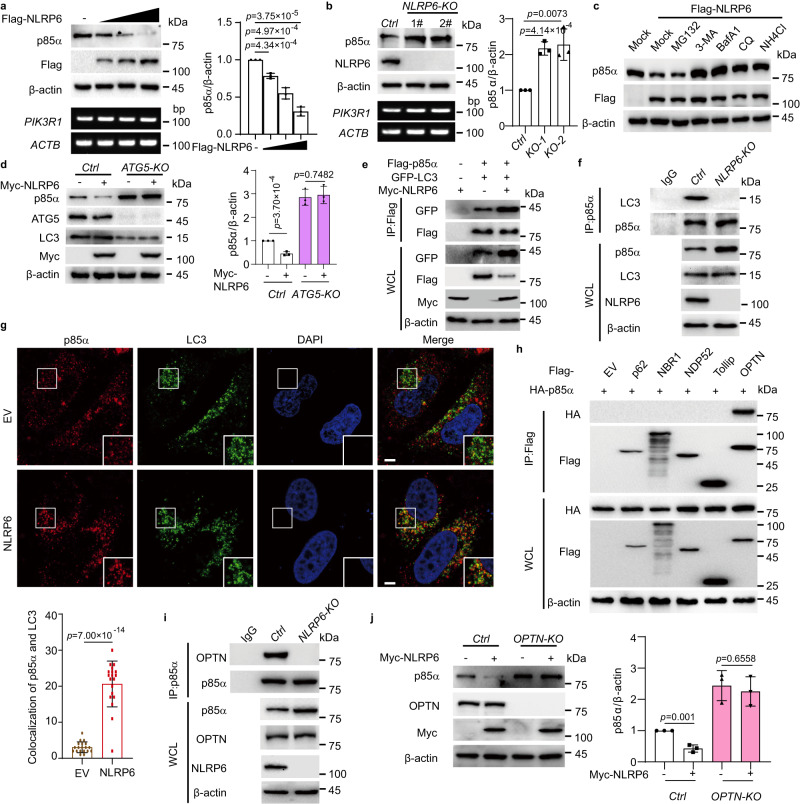
NLRP6 promotes p85α autophagic degradation via the cargo receptor OPTN. **a** p85α protein (top) and *PIK3R1* mRNA (bottom) levels were analysed in LN229 cells transfected with increasing amounts of Flag-NLRP6. p85α protein levels were quantified. **b** p85α protein (top) and *PIK3R1* mRNA (bottom) levels were analysed in Control (*Ctrl*) and *NLRP6* knockout (KO) LN229 cells. p85α protein levels were quantified. **c** LN229 cells transfected with Flag-NLRP6 were treated with DMSO (Mock), MG132, 3-methyladenine (3-MA), bafilomycin A1 (BafA1), chloroquine (CQ), or NH_4_Cl, and the effects on p85α protein levels were examined. **d** p85α protein levels were analysed in *Ctrl* and *ATG5* KO LN229 cells and were quantified. **e** Coimmunoprecipitation (Co-IP) analysis to investigate the interaction between p85α and LC3 in the presence of NLRP6 in HEK293T cells. WCL, whole cell lysates. **f** Co-IP analysis of the interaction between endogenous p85α and LC3 in the absence of *NLRP6* in LN229 cells. **g** Representative confocal microscopy of LN229 cells transfected with NLRP6. Scale bar = 5 μm (top). Statistical analysis of the number for p85α and LC3 colocalization (bottom). **h** Co-IP analysis to map the interaction between p85α and cargo receptors in HEK293T cells. **i** Co-IP analysis of the interaction between p85α and OPTN in the absence of *NLRP6* in LN229 cells. **j** p85α protein levels were analysed in *Ctrl* and *OPTN* KO LN229 cells transfected with Myc-NLRP6 (left) and were quantified (right). In **a**, **b**, **d** and **j**, all error bars, mean values ± SD, *p*-values were determined by unpaired two-tailed Student’s *t* test of *n* = 3 independent biological experiments. For **g**, error bar, mean value ± SD, *p*-value were determined by unpaired two-tailed Student’s *t* test of *n* = 19 cells. For **c**, **e**, **f**, **h**, and **i**, data shown are representative of three independent experiments with similar results. Source data are provided as a Source Data file.

Class I PI3K is a heterodimer which is composed by one p110 catalytic subunit and one p85 regulatory subunit^1^. As the regulatory subunit p85α was found to interact with NLRP6, we wondered the relationship between NLRP6 and the subunits of PI3K. We found that NLRP6 could directly interact with p85α, but not p55γ, p85β, p110γ, p110δ (Supplementary Fig. 3a). In addition, NLRP6 could only promote the degradation of p85α, but not p55γ, p85β, p110γ, or p110δ (Supplementary Fig. 3b). These results suggested that NLRP6 could specifically interact with p85α to reduce its protein level, but not other subunits of PI3K. In order to investigate the molecular mechanisms by which NLRP6 decreased the protein abundance of endogenous p85α, we transfected increasing amounts of Flag-NLRP6 into LN229 cells and found that the endogenous p85α protein level was gradually decreased while the *PIK3R1* mRNA level was barely affected, indicating that NLRP6 controlled the protein stability of p85α (Fig. 2a and Supplementary Fig. 3c). In contrast, the endogenous p85α protein level, but not its mRNA level, was significantly increased in *NLRP6* KO cells (Fig. 2b and Supplementary Fig. 3d). The autophagy-lysosome pathway (ALP) and the ubiquitin-proteasome system (UPS) are the two major degradation systems for cellular proteostasis^23^. To determine which degradation system may dominantly regulate the degradation of p85α mediated by NLRP6, we examined protein stability of p85α using different pharmacologic approaches. We found that the autophagy and lysosome inhibitors 3-methyladenine (3-MA), bafilomycin A1 (BafA1), chloroquine (CQ), and NH_4_Cl, but not the proteasome inhibitor MG132, attenuated NLRP6-induced p85α degradation, indicating that NLRP6-mediated p85α degradation was predominantly mediated through the ALP system (Fig. 2c). Autophagy is a tightly controlled and balanced biological process. ATG5 is one of the key players in autophagy and it is involved in the formation of the autophagosome in both canonical and noncanonical autophagy processes^24^. p85α degradation induced by NLRP6 overexpression was almost abrogated in *ATG5* deficient cells, further indicating that p85α degradation mediated by NLRP6 was mainly dependent on autophagic degradation (Fig. 2d). LC3 is specifically ligated with autophagosomes and is a widely used marker for autophagosomes^25^. We next observed that NLRP6 overexpression promoted the interaction between p85α and LC3 (Fig. 2e), while *NLRP6* KO showed the opposite effect (Fig. 2f and Supplementary Fig. 3e). The colocalization of p85α and LC3 were significantly increased in the presence of NLRP6, while global LC3 puncta formation was barely influenced (Fig. 2g), indicating that NLRP6 might promote p85α degradation through selective autophagy, which is specifically regulated by cargo receptors^26^. Concordantly, we found that p85α specifically interacts with the cargo receptor OPTN (Optineurin, encoded by *Optineurin*) (Fig. 2h). In addition, we demonstrated that the interaction between p85α and OPTN was fully dependent on NLRP6 (Fig. 2i and Supplementary Fig. 3f). We further showed that p85α degradation induced by NLRP6 overexpression was almost blocked in *OPTN* KO cells, indicating the important role of OPTN in NLRP6-mediated p85α degradation (Fig. 2j). Taken together, these results suggested that NLRP6 promotes autophagic degradation of p85α via the cargo receptor OPTN.

### K245 and K256 on p85α are critical for its ubiquitination and degradation

**Fig. 3:**
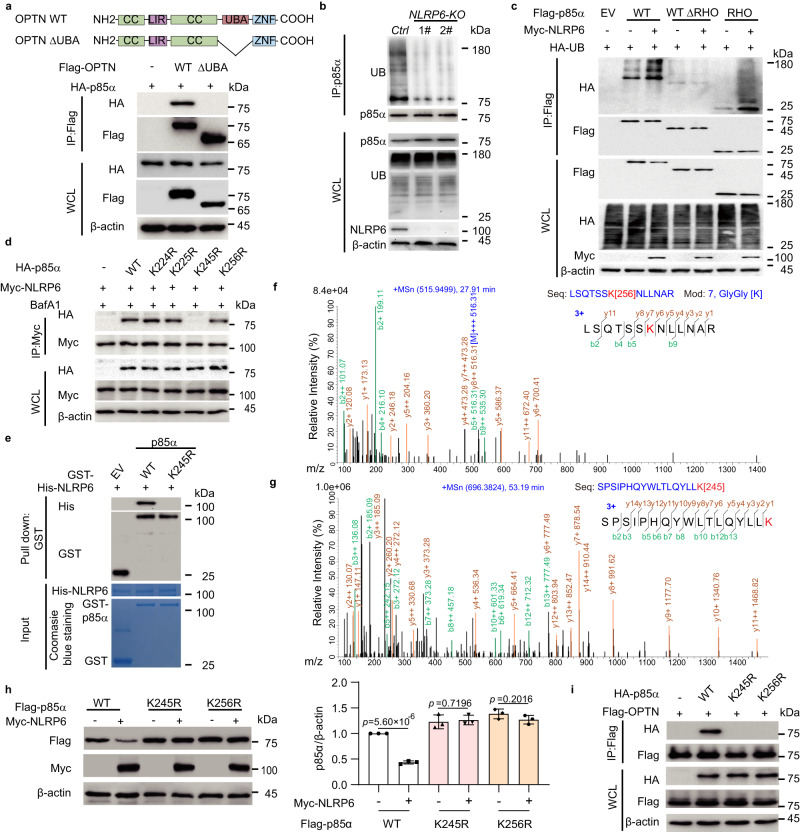
Ubiquitination of p85α on K256 is critical for autophagic degradation. **a** Coimmunoprecipitation (Co-IP) and immunoblot analysis of extracts of LN229 cells transfected with HA-p85α together with Flag-tagged wild type (WT) OPTN or ΔUBA-OPTN. WCL, whole cell lysates. **b** Co-IP and immunoblot analysis of extracts from Control (*Ctrl*) or *NLRP6* knockout (KO) LN229 cells. **c** Co-IP and immunoblot analysis of extracts of HEK293T transfected with HA-UB and empty vector (EV), Flag-p85α (WT), Flag-p85α (ΔRHO), or Flag-p85α (RHO) in the presence of Myc-NLRP6. **d** Co-IP and immunoblot analysis of extracts of LN229 cells transfected with Myc-NLRP6 and empty vector (EV), HA-p85α (WT), HA-p85α (K224R), HA-p85α (K225R), HA-p85α (K245R), or HA-p85α (K256R) in the presence of BafA1 (0.2 μM). **e** Co-IP and immunoblot analysis of purified His-NLRP6 with GST-p85α (WT) or GST-p85α (K245R). **f** Mass spectrometry analysis of a peptide derived from p85α identifying the attachment of ubiquitin to K256. **g** Mass spectrometry analysis of a peptide derived from p85α identifying that ubiquitin was not attached to K245. **h** Immunoblot analysis of extracts of LN229 cells transfected with Flag-p85α (WT), Flag-p85α (K245R), and Flag-p85α (K256R) in the presence of Myc-NLRP6 (left). Quantification of the protein levels of p85α (right). **i** Co-IP analysis of the interaction between various p85α mutants and OPTN in HEK293T cells. In **h**, all error bars, mean values ± SD, *p*-values were determined by unpaired two-tailed Student’s *t* test of *n* = 3 independent biological experiments. Data are representative of three independent experiments with similar results (**a**, **b**, **d**, **e**, and **i**), or two independent experiments (**c**). Source data are provided as a Source Data file.

During selective autophagy, ubiquitin chains attached to substrates are recognized by cargo receptors through their own ubiquitin-associated (UBA) domains^27^. We observed that p85α could interact with OPTN but not its ΔUBA mutant (Fig. 3a), indicating that NLRP6-mediated p85α degradation requires ubiquitin chains as the recognition signal for OPTN binding. We next investigated the effect of NLRP6 on the ubiquitination status of p85α, and found that *NLRP6* deficiency significantly attenuated p85α ubiquitination (Fig. 3b). The RHO domain on p85α was significantly ubiquitinated in the presence of NLRP6 while p85α ΔRHO mutant was not, suggesting that the RHO domain on p85α was required for ubiquitination (Fig. 3c). To further identify the specific lysine (K) residues to which ubiquitin was covalently attached, we identified eight conserved key lysine residues (K134, K141, K142, K187, K224, K225, K245, and K256) in the RHO domain across species (Supplementary Fig. 4a). We then mutated each lysine to arginine (R) to generate the corresponding p85α mutants bearing a single K-to-R substitution (Supplementary Fig. 4b). Compared with WT p85α, the p85α mutants bearing K245R or K256R attenuated ubiquitin (UB) conjugation on p85α when NLRP6 was introduced, indicating that K245 and K256 on p85α were critical for NLRP6-mediated p85α ubiquitination (Supplementary Fig. 4c). We next analysed the interaction between NLRP6 and p85α mutants (K224R, K225R, K245R, and K256R). Interestingly, the K245R mutation but not the K256R mutation can completely block the interaction between p85α and NLRP6 (Fig. 3d), suggesting K245 may be the interaction site of NLRP6 but not the ubiquitination site. To test our hypothesis, we purified p85α K245R mutant and found that it could not bind with NLRP6 in vitro (Fig. 3e), suggesting that K245 is required for the NLRP6-p85α interaction. To further identify the ubiquitination site of p85α, we isolated ubiquitinated forms of Flag-p85α for multidimensional liquid chromatography (LC) followed by mass spectrometric (MS) analysis (Supplementary Fig. 4d). We found that the ubiquitin moieties were on residue K256 but not on residue K245, further confirming that K245 is an interacting site of NLRP6 rather than the ubiquitination site on p85α (Figs. 3f, g). In *NLRP6* KO cells, the ubiquitination signal of p85α on K256 could not be detected in p85α immunoprecipitates. In addition, no other ubiquitination sites on RHO domain were detected through the LC-MS method. We further showed that the protein degradation induced by NLRP6 was completely blocked in the p85α K245R and K256R mutants (Fig. 3h). Finally, the direct association between p85α and OPTN also disappeared when K245 or K256 was mutated on p85α (Fig. 3i). As K245 on p85α was the critical residue for its interaction with NLRP6 and K256 on p85α was the critical ubiquitination site for p85α autophagic degradation, mutation of either of these two sites could disrupt the association between p85α and OPTN. Taken together, these results suggested that K245 on p85α is the critical site for the direct interaction between p85α and NLRP6, which allows NLRP6 to mediate the ubiquitination of p85α at K256.

### NLRP6 recruits RBX1 to ubiquitinate p85α

**Fig. 4:**
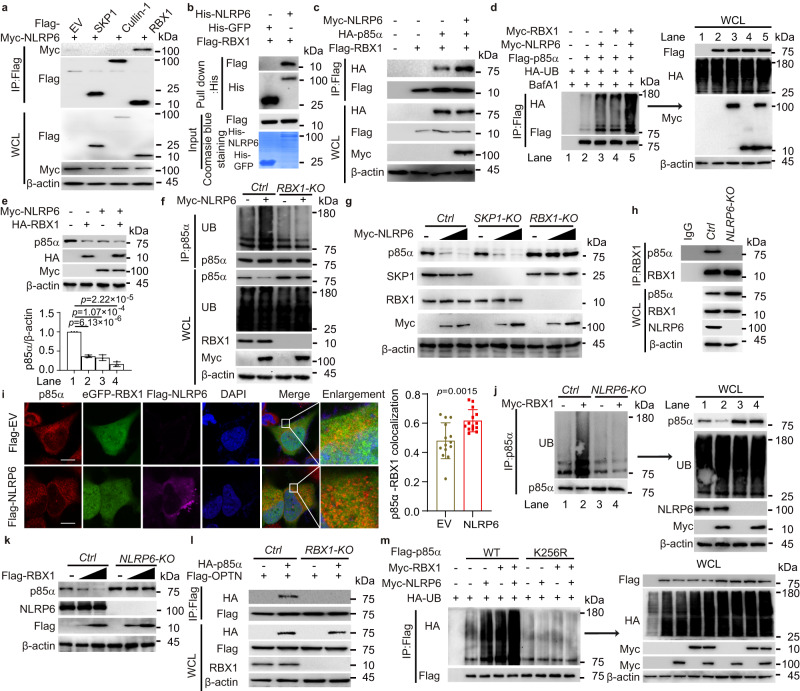
NLRP6 recruits RBX1 to ubiquitinate p85α. **a** Coimmunoprecipitation (Co-IP) and immunoblot analysis of HEK293T cells transfected with Myc-NLRP6 and indicated plasmids. EV, empty vector, WCL, whole cell lysates. **b** Co-IP analysis of the interaction between NLRP6 and RBX1. **c** Co-IP analysis of the interaction between p85α and RBX1 in the presence of NLRP6 in HEK293T cells. **d** p85α-associated ubiquitins (UBs) were analysed in HEK293T cells transfected indicated plasmids with bafilomycin A1 (BafA1, 0.2 μM). **e** Endogenous p85α in LN229 cells transfected with indicated plasmids were analysed (top) and were quantified (bottom). **f** p85α-associated UBs were analysed in Control (*Ctrl*) or *RBX1* knockout (KO) LN229 cells transfected with Myc-NLRP6. **g** Endogenous p85α in *Ctrl*, *SKP1* KO, or *RBX1* KO LN229 cells transfected with increasing amounts of Myc-NLRP6 were analysed. **h** Co-IP analysis of the interaction between p85α and RBX1 in *Ctrl* and *NLRP6* KO LN229 cells. **i** Representative confocal microscopy of LN229 cells transfected with NLRP6 showing p85α and RBX1 colocalization (left). Scale bar = 10 μm. Pearson’s coefficient of colocalization was calculated (right). **j** p85α-associated UBs in *Ctrl* or *NLRP6* KO LN229 cells transfected with Myc-RBX1 were analysed. **k** Endogenous p85α in *Ctrl* or *NLRP6* KO LN229 cells transfected with increasing amounts of Flag-RBX1 were analysed. **l** Co-IP and immunoblot analysis of extracts from *Ctrl* or *RBX1* KO LN229 cells transfected with indicated plasmids. **m** Co-IP and immunoblot analysis of LN229 cells transfected with indicated plasmids. In e, all error bars, mean values ± SD, *p*-values were determined by unpaired two-tailed Student’s *t* test of *n* = 3 independent biological experiments. For **i**, error bar, mean value ± SD, *p*-value were determined by unpaired two-tailed Student’s *t* test of *n* = 14 cells. Data are representative of three independent experiments with similar results (**a**–**d**, **f**, **h**, and **j**–**m**), or two independent experiments (**g**). Source data are provided as a Source Data file.

As NLRP6 is not an E3 ubiquitin ligase, we reasoned that NLRP6 may function as an adaptor to recruit certain E3 ubiquitin ligase to p85α. Among the candidates interacting with NLRP6 through the MS data, SKP1 and RBX1, which are critical components of the SCF (SKP1/Cullin-1/F-box protein) complex, caught our attention (Supplementary Fig. 5a). Although Cullin-1 did not appear in the candidate list, we added Cullin-1 in the following experiments, as it is another important component in the SCF complex^28^. We next checked the physical association of NLRP6 with SKP1, Cullin-1 or RBX1 and found that NLRP6 was directly associated with RBX1 but not with SKP1 or Cullin-1 (Figs. 4a, b). As NLRP6 bound with p85α and RBX1, we speculated that NLRP6 could bridge RBX1 to p85α for ubiquitination. Indeed, we found that NLRP6 prompted the interaction between p85α and RBX1 (Fig. 4c). In the SCF complex, Cullin-1 is a scaffold component that serves as the assembly centre, and RBX1/2 is the catalytic RING component that transfers ubiquitin from E2 enzymes to target substrates^29^. This result inspired us to conclude that NLRP6 might act as a scaffold protein with a function similar to that of Cullin-1 and recruit RBX1 to ubiquitinate p85α. We showed that NLRP6 alone or RBX1 alone could induce the attachment of ubiquitin to p85α in LN229 cells, but the combination of NLRP6 and RBX1 could further enhance this process (Fig. 4d). The effect of NLRP6 and RBX1 on p85α protein stability was also determined. As an E3 ubiquitin ligase, RBX1 could promote p85α degradation in a dose-dependent manner (Supplementary Fig. 5b), while the combination of NLRP6 and RBX1 could further enhance this phenomenon (Fig. 4e). We next sought to identify whether the destabilization of p85α induced by NLRP6 was dependent on RBX1 and observed that NLRP6-directed ubiquitination and degradation of p85α were almost abolished in *RBX1* KO cells (Figs. 4f, g and Supplementary Fig. 5c). Furthermore, we found that RBX1 could no longer bind with p85α in *NLRP6* KO cells (Fig. 4h and Supplementary Fig. 5d). The colocalization of p85α and RBX1 was significantly increased when NLRP6 was overexpressed (Fig. 4i). Enhanced ubiquitination (Fig. 4j) and degradation (Fig. 4k) of p85α induced by RBX1 were also abolished in *NLRP6* KO cells. Collectively, these results suggested that both NLRP6 and RBX1 were required for the control of p85α ubiquitination and degradation. Consistent with these data, OPTN no longer recognized p85α in *RBX1* KO cells (Fig. 4l). The ubiquitination of p85α induced by NLRP6 and RBX1 was almost abolished when K256 on p85α was mutated to R256 (Fig. 4m), suggesting that NLRP6-RBX1 catalysed the ubiquitination of p85α at K256. In order to confirm that NLRP6-RBX1 is a distinct E3 ligase complex which is different from the conventional Cullin-RBX1 ubiquitin E3 ligase complex, the interaction between RBX1 and Cullin-1 or NLRP6 was investigated. RBX1 was able to coimmunoprecipitate with endogenous NLRP6 or Cullin-1 respectively, while NLRP6 could not interact with Cullin-1 (Supplementary Fig. 5e). Furthermore, when *CUL-1* was knocked out, NLRP6 could still interact with RBX1 (Supplementary Fig. 5f) and promote ubiquitination (Supplementary Fig. 5g) and degradation (Supplementary Fig. 5h) of p85α. Taken together, these results demonstrated that NLRP6 could act as the structural scaffold of the NLRP6-RBX1-p85α complex and recruit RBX1 to ubiquitinate p85α, thus leading to the autophagic degradation of p85α with the assistance of OPTN.

### NLRP6 promotes glioma tumorigenesis

**Fig. 5:**
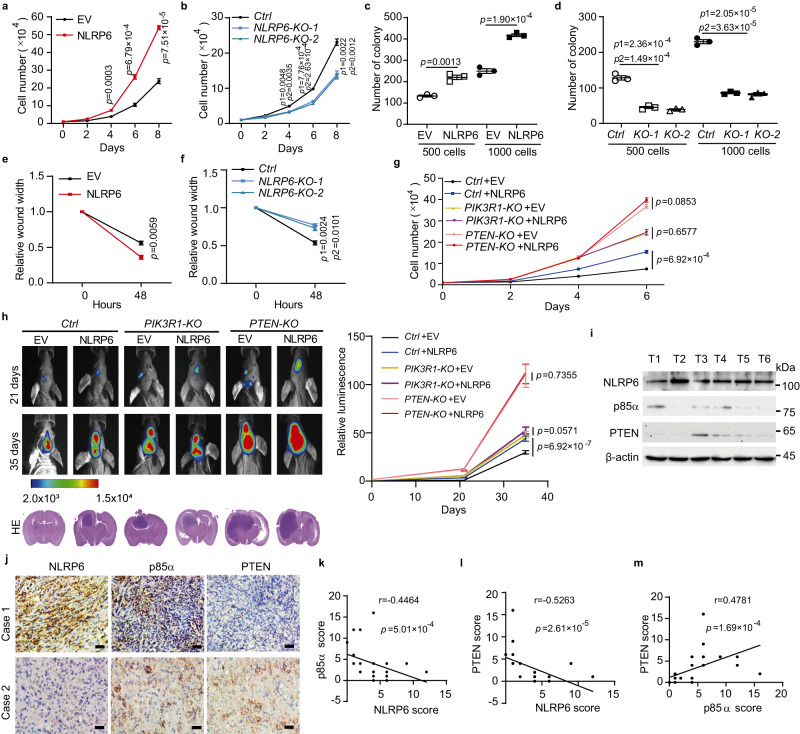
NLRP6 promotes glioma tumorigenesis. **a** Cell proliferation assay of NLRP6-overexpressing LN229 cells compared with empty vector (EV)-transfected cells. **b** Cell proliferation assay of *NLRP6* knockout (KO) LN229 cells compared with control cells (*Ctrl*). **c** Colony formation assay of NLRP6-overexpressing LN229 cells compared with EV. **d** Colony formation assay of *NLRP6* KO LN229 cells compared with *Ctrl*. **e** Cell wound healing assay of NLRP6-overexpressing LN229 cells compared with EV. **f** Cell wound healing assay of *NLRP6* KO LN229 cells compared with *Ctrl*. **g** Cell proliferation assay of *Ctrl*, *PIK3R1* KO, or *PTEN* KO LN229 cells transfected with EV or NLRP6 plasmid. **h** In vivo bioluminescence imaging of nude mice with intracranially implanted *Ctrl*, *PIK3R1* KO, or *PTEN* KO LN229 cells transfected with EV or NLRP6 plasmid. Representative bioluminescence images (left) and quantitative analysis of relative bioluminescence are shown (right). **i** Immunoblot analysis of NLRP6, p85α, and PTEN protein expression in GBM tissues. **j** Immunohistochemistry (IHC) analysis of NLRP6, p85α, and PTEN in GBM tissue paraffin sections. Scale bar = 50 μm. **k** Correlation between NLRP6 and p85α IHC scores. **l** Correlation between NLRP6 and PTEN IHC scores. **m** Correlation between p85α and PTEN IHC scores. *p*1 or *p*2 was the *p*-value for comparing *NLRP6-KO-1* or *NLRP6-KO-2* with *Ctrl*. In a-g, all error bars, mean values ± SEM, *p*-values were determined by unpaired two-tailed Student’s *t* test of *n* = 3 independent biological experiments. In **h**, all error bars, mean values ± SD, *p*-values were determined by unpaired two-tailed Student’s *t* test (*n* = 6 mice per group). For **i**, data shown are representative of three independent experiments with similar results. For **k**–**m**, *p*-values are determined by two-sided Pearson’s correlation test. Source data are provided as a Source Data file.

To comprehensively understand the role of NLRP6 in tumour progression, we investigated the biological function of NLRP6. NLRP6 overexpression significantly enhanced cell proliferation, colony formation, and cell migration, while *NLRP6* deficiency exerted the opposite effects in LN229 cells (Figs. 5a–f), LN18 cells (Supplementary Figs. 6a–f), and HS683 cells (Supplementary Figs. 6g–l). In contrast, NLRP6 level did not affect cell proliferation in U251 cells, in which PTEN was deficient^20^ (Supplementary Figs. 6m, n). Ectopic p85α significantly suppressed cell proliferation, while its loss exerted the opposite function in LN229 cells (Supplementary Figs. 6o, p). Consistently, p85α did not affect cell proliferation in PTEN deficient U251 cells (Supplementary Figs. 6o, p). Furthermore, NLRP6 could not promote cell proliferation in *PTEN* KO or *PIK3R1* KO cells (Fig. 5g). To investigate the biological function of NLRP6 in vivo, we intracranially injected the indicated LN229 cells into nude mice to monitor the progression of brain tumours. Consistent with the in vitro results, we found that NLRP6 overexpression promoted glioma progression (Fig. 5h), while *NLRP6* deficiency inhibited tumour growth in vivo (Supplementary Fig. 6q). Furthermore, NLRP6-induced glioma progression was abolished when *PTEN* or *PIK3R1* was deficient (Fig. 5h).

We next analysed the expression levels of NLRP6, p85α, and PTEN in human GBM tissues. Due to the reasons that NLRP6 controlled p85α stability at the protein level and it is hard to find NLRP6 and p85α protein expression data in GBM tissues in public database, we chose to use our own samples. PCR-SSCP analysis was performed to determine the *PTEN* status in GBM samples. The inclusion criteria were as follows: (1) newly diagnosed GBM and (2) PTEN WT. In total, 57 GBM samples were selected. The protein levels of NLRP6, p85α, and PTEN were detected by immunoblot in GBM tissue samples (Fig. 5i) and by immunohistochemistry in paraffin sections (Fig. 5j). The results supported our hypothesis that when NLRP6 was upregulated, p85α and PTEN were downregulated in GBM tissues. The correlation between NLRP6, p85α, and PTEN in GBM tissues was also evaluated by Pearson’s correlation assay. NLRP6 was negatively correlated with p85α (Fig. 5k) and PTEN (Fig. 5l), while p85α was positively correlated with PTEN (Fig. 5m). Taken together, these results suggested that NLRP6 acts as an oncogene in glioblastoma.

### Disturbing NLRP6/p85α interaction inhibits tumour growth

**Fig. 6:**
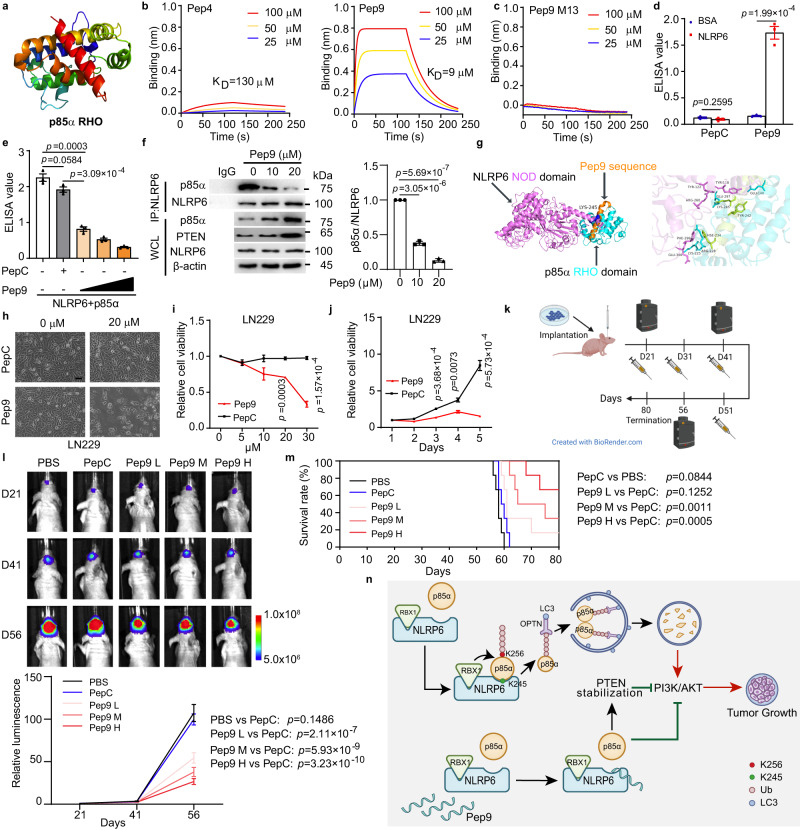
Blockage of NLRP6/p85α interaction inhibits tumour growth. **a** Crystal structure of the p85α RHO domain. **b** Biolayer interferonmetry (BLI) analysis of kinetic interactions between Pep4 or Pep9 and recombinant NLRP6. **c** BLI analysis of kinetic interaction between Pep9 M13 mutant and NLRP6. **d** ELISA analysis of binding ability of HRP-conjugated PepC and HRP-conjugated Pep9 to BSA or purified NLRP6. **e** ELISA showing competition of NLRP6 binding to p85α with PepC or Pep9. **f** NLRP6-associated p85α were analysed in LN229 cells after treatment with TAT-Pep9 (left) and were quantified (right). **g** The overall structure of the complex (left). Schematic diagram of complex interaction residues (right). The residues on the NLRP6 NOD domain are shown in violet, on Pep9 are shown in limon, and on the p85α RHO domain but not on Pep9 are shown in cyan. **h** LN229 cells were treated with TAT-PepC or TAT-Pep9 for 24 h. Scale bar = 50 μm. **i** Cell proliferation assay of LN229 cells treated with different concentrations of TAT-Pep9. **j** Cell proliferation assay of LN229 cells treated with TAT-Pep9 at the indicated time points. **k** Experimental timeline of mice tumour model with peptide treatment. **l** Representative bioluminescence images (top) and quantitative analysis (bottom) of relative bioluminescence from mice after different treatments. **m** Survival analysis of nude mice after different treatments. **n** Schematic diagram. In **d**, **e**, **i**, and **j**, all error bars, mean values ± SEM, *p*-values were determined by unpaired two-tailed Student’s *t* test of *n* = 3 independent biological experiments. In **f**, all error bars, mean values ± SD, *p*-values were determined by unpaired two-tailed Student’s *t* test of *n* = 3 independent biological experiments. In l data show values ± SD, unpaired two-tailed Student’s *t* test (*n* = 6 mice per group). For **b**, **c**, and **h**, data shown are representative of three independent experiments with similar results. For **m**, *p*-values were determined using a log-rank (Mantel–Cox) test. Source data are provided as a Source Data file.

Protein-protein interactions are emerging as new potential therapeutic targets^30^. We demonstrated that NLRP6 promoted tumour growth through its direct interaction with p85α to promote p85α degradation. Thus, interrupting the interaction between NLRP6 and p85α appears to be an attractive strategy to inhibit tumour growth. α-Helices are the fundamental recognition elements in protein-protein interactions and may serve as ideal inhibitors of macromolecular interactions^30^. According to the observations that the SR domain (amino acids 1–301) on p85α interacted with the NOD domain (amino acids 104–726) on NLRP6 (Figs. 1e, f) and that the RHO domain was ubiquitinated by NLRP6 (Fig. 3c), it was possible to screen the α-helical peptides based on the p85α secondary structure and to identify which might disturb the NLRP6/p85α interaction. Due to the lack of the crystal structure of the p85α SH3 domain, the structure of the human p85α SH3 domain was built by using I-TASSER to search for potential α-helical peptides^31^. Two peptides (Pep1 and Pep2) were found on the p85α SH3 domain. The 3D structure of the human p85α RHO domain was extracted from the crystal structure in the Protein Data Bank (ID: 1PBW) (Fig. 6a). Nine peptides (Pep3-Pep11) were found on the p85α RHO domain. The binding affinity (K_D_) of each peptide to recombinant NLRP6 was measured using a biolayer interferometry (BLI) assay. Among them, the peptides Pep4 and Pep9 could bind with NLRP6 (Fig. 6b), while others could not (Supplementary Fig. 7). Moreover, Pep9 displayed a much higher affinity for NLRP6 than Pep4. It was demonstrated that the K245 residue on p85α was critical for the NLRP6-p85α interaction, and it was surprising to find that the residue was on Pep9 at position 13. To decipher whether the critical amino acid residue might determine the binding affinity of Pep9 for NLRP6, the amino acid residue of Pep9 was substituted with alanine (A), and the binding affinity of the Pep9 mutant for NLRP6 was measured. Compared with Pep9, Pep9 M13 dramatically abolished the binding affinity of Pep9 with NLRP6 (Fig. 6c). As Pep1 did not interact with NLRP6 (Supplementary Fig. 7a), Pep1 was selected as the control named PepC in the following experiments. Horseradish peroxidase (HRP)-conjugated Pep9, but not HRP-conjugated PepC, could bind to NLRP6 (Fig. 6d). In addition, Pep9 inhibited the binding of p85α to NLRP6 in a dose-dependent manner (Fig. 6e). Next, we determined whether the Pep9 peptide could disturb the NLRP6/p85α interaction. The cell penetrating peptide (CPP) TAT (GGRKKRRQRRR) is a commonly used short peptide for drug delivery in glioma. The CPP TAT was linked to Pep9 to facilitate Pep9 intracellular delivery. TAT-Pep9 significantly decreased the interaction between NLRP6 and p85α in a dose-dependent manner (Fig. 6f). With the information obtained from our experiments, CoDockPP was applied to predict the complex structure of the p85α RHO domain interacting with the NLRP6 NOD domain^32^. The binding mode of the complex showed that the loop region of the p85α RHO domain inserts into the pocket of the NLRP6 NOD domain (Fig. 6g, left). The predicted complex structure indicated that residues TYR118, GLU300, PHE299, TYR122, and ARG260 in the NLRP6 NOD domain interacted with residues GLU140, LYS225, ARG228, HSE234, TYR242, LYS245, and GLU297 in the p85α RHO domain with interaction energy contributions lower than −8.0 kcal/mol, suggesting that these residues were important for the interaction between the p85α RHO domain and NLRP6 NOD domain. Among the 7 residues on the p85α RHO domain that were predicted to interact with NLRP6, 3 residues were located on Pep9 (Fig. 6g, right). SC-79 specifically binds to the PH domain of AKT and activates AKT in the cytosol^33^. The inhibition of Pep9 on PI3K/AKT pathway was reversed when SC79 was added (Supplementary Fig. 8a). Next, the antitumour efficacy of Pep9 was evaluated in vitro and in vivo. TAT-Pep9 significantly inhibited cell proliferation in a dose-dependent and time-dependent manner in LN229 cells (Figs. 6h–j) as well as in LN18 (Supplementary Figs. 8b–d) and HS683 cells (Supplementary Figs. 8e–g), but not in *PTEN* deficient U251 cells (Supplementary Figs. 8h–j). According to our preliminary experiment, TAT-Pep9 could barely enter the nude mouse brain through intravenous injection or intraperitoneal injection. Thus, the intratumoural delivery method was selected. Consistent with the in vitro results, TAT-Pep9 treatment could also enhance the abundance of p85α protein (Supplementary Fig. 8k) and inhibit tumour growth in vivo (Figs. 6k, l). Furthermore, TAT-Pep9 could prolong the survival of these animals, especially at a high dose (Fig. 6m). Taken together, these results suggested that interrupting the NLRP6/p85α interaction by Pep9 produces potent antitumour efficacy.

In conclusion, our studies demonstrate that NLRP6 potentiates PI3K/AKT pathway by promoting p85α degradation via selective autophagy and that targeting the NLRP6/p85α interaction might serve as a promising therapeutic strategy to inhibit PI3K/AKT pathway against tumour in future (Fig. 6n).

## Discussion

NOD-like receptors (NLRs) are widely known to mediate the host innate immune response to cellular stress in different physiological processes^34^. Increasing evidence has extended the concept that NLRs not only function as PRRs to recognize PAMPs to initiate immune responses, but also participate in tumorigenesis. Here, we demonstrated that NLRP6 acts as a scaffold protein to interact with p85α and recruit RBX1 to ubiquitinate p85α for OPTN-mediated autophagic degradation, leading to PI3K/AKT activation to enhance tumour progression. Disruption of the NLRP6/p85α interaction could stabilize p85α, inhibit the PI3K/AKT pathway, and suppress tumour growth, indicating a promising therapeutic strategy against glioma.

NLRP6 is a multifaceted innate immune sensor with physiological functions ranging from microbiota-epithelial crosstalk^35^, host defence against pathogens^36,37^, and metabolic diseases^38^ to neuroinflammation^39^ in mediating the initial innate immune response to different cellular stresses^40^. During these processes, NLRP6 is known to regulate caspase-1, nuclear factor-κB (NF-κB), and mitogen-activated protein kinase (MAPK) signalling pathways^41^. NLRP6 inflammasome plays an important role in the maintenance of epithelial integrity and host defence against microbial infections^42^. In the presence of a specific stimuli, NLRP6 inflammasome is assembled followed by caspase-1 activation, which cleaves pro-IL-18 and pro-IL-1β into their active forms, leading to enhanced IFN-γ, TNF-α, IL-17, and IL-6 expression as well as cell pyroptosis^40,42^. The activation of NLRP6 inflammasome induced by liquid-liquid phase separation of NLRP6 requires specific stimulations such as RNA virus infection^43^. Considering that all our experiments were performed under normal conditions without any stimulation such as microbial infections, the effects of NLRP6 on p85α and its downstream signalling pathway are independent of NLRP6 inflammasome activation. In addition to its roles in innate immunity, NLRP6 is also implicated in carcinogenesis. For example, NLRP6 was reported to control epithelial self-renewal and microbiota homoeostasis to protect against inflammation-induced tumour development in the intestine^44–46^, although the finding that NLRP6 exacerbated graft-versus-host disease, which was independent of microbiome change, was in contrast to the protective role of NLRP6 in tumorigenesis^47^. Unlike the components that take part in the formation of the NLRP6 inflammasome, these specific molecules play important roles in tumour development through their direct interaction with NLRP6, such as DHX15^48^ in the intestine, GRP78 in gastric cancer^49^, and p85α in glioma in our study. In the present study, NLRP6 protein level is a rate-limiting factor for p85α degradation in glioma. NLRP6 acts as a threshold to control PI3K/AKT pathway activation by promoting p85α autophagic degradation. The increase of NLRP6 expression could reduce p85α protein level and enhance PI3K/AKT pathway activation, leading to glioma tumorigenesis. Thus, the upregulation of NLRP6 might facilitate tumorigenesis. In consistent with this hypothesis, NLRP6 expression level is negatively correlated with p85α expression level in human glioblastoma samples. These findings further support that NLRP6 participates in various physiological and pathological processes in different contexts through different molecular interactions. The different effects of NLRP6 on tumour growth depend on tissue specificity, the tumour microenvironment, and, most importantly, the molecules with which NLRP6 directly interacts under specific physiological conditions.

RBX1 is the key ubiquitin E3 ligase in NLRP6-induced p85α ubiquitination. RBX1 is the catalytic RING component of the SCF (SKP1/Cullin-1/F-box proteins) complex, which forms the largest family of E3 ubiquitin ligases and promotes the ubiquitination of diverse cellular proteins in multiple physiological processes^50^. Usually, the specificity of the SCF complex is determined by the F-box protein that bridges the SCF components and its target protein, while the ligase activity of the SCF complex is determined by the Cullin-RBX complex, which transfers ubiquitin from RBX-bound E2 to the target protein^51^. The central dogma of RBX1 function is that RBX1 can heterodimerize with Cullin and therefore form the Cullin-directed ubiquitin E3 ligase complex to trigger substrate protein ubiquitination^52^. In the present study, we demonstrated that NLRP6 could act as a scaffold protein, to recognize its protein substrate p85α, and help RBX1 transfer ubiquitin to the lysine residues on p85α. NLRP6 can act similarly to Cullin to trigger ubiquitination of its interaction protein p85α, which unveils the distinct nature of NLRP6-RBX1 as an alternative E3 ligase complex, differing from the conventional Cullin-RBX1 ubiquitin E3 ligase complex. As there were over four hundred potential candidate proteins which could interact with NLRP6, we speculate that besides NLRP6 and RBX1, there are other proteins which could also regulate p85α stability under different physiological conditions in a NLRP6-independent pathway. In addition, we identified OPTN as the cargo receptor responsible for NLRP6-mediated p85α degradation. Among several main selective cargo receptors, OPTN was the only one which could interact with p85α. The autophagic cargo receptors specifically bind the cargo materials and recruit them to autophagosomes, leading to protein autophagic degradation^53^. Without the direct interaction between the cargo receptor and its targeted components, the autophagic degradation programme could not be preceded. Our research just unveils a small part of the complex protein network and more work should be done to comprehensively demonstrate the regulation of p85α protein stability.

Protein-protein interactions are one of the most fundamental interactions in almost all biological processes, and their dysregulation is the signature of many diseases^54^. Molecules that may specifically disrupt these interactions are promising candidates for medical diagnosis and therapeutic intervention^54^. Recent studies have provided evidence that α-helical peptides based on the interface of two or more proteins hold promise for inhibiting protein-protein interactions^55^. For example, the TAT-TROY peptide disrupts the interaction between TROY and RKIP and reduces glioma development in vitro and in vivo^56^. TAX2 specifically inhibits the THBS1/CD47 interaction and decreases cell invasion in GBM^57^. In the present study, the peptide Pep9 disrupted the direct interaction between p85α and NLRP6 followed by p85α stabilization and diminished PI3K/AKT activity, highlighting its potential to be developed as a peptide drug. With advances in structural biology, recombinant technology, and synthetic technology, an increasing number of new drugs based on peptides have emerged in the last 50 years^58^. Although peptide drugs have advantages such as lower immunogenicity, lower cost in large production, and more accurate targeting efficiency on “undruggable” protein-protein interactions when compared with small molecule drugs, peptide drugs also have their disadvantages: 1) Peptides have relatively weak cell membrane permeability due to the long length of the peptide and the composition of amino acids. In the present study, the cell penetrating peptide TAT was linked to Pep9 for its delivery into cancer cells. 2) Peptides have poor stability in vivo. The peptides are highly unstable and easily degraded under physiological conditions as the amide bonds are recognized and hydrolysed by multiple enzymes in vivo. The peptide stability can be enhanced by chemical modification, L-amino acid substitution, methyl-amino acid insertion or other new emerging technologies^59^. In addition, it is difficult for peptide to penetrate through the blood brain barrier into the central nervous system. Although the intratumoural delivery method was used in our experiment, this strategy will limit its future use in clinical practice. With advances in peptide design, synthetic methodology, and drug delivery technology, peptide disadvantages will be overcome, and peptide drug development will enter a new era.

In conclusion, our studies demonstrate that NLRP6 acts as a scaffold protein to interact with p85α and recruit RBX1 to ubiquitinate p85α for OPTN-mediated autophagic degradation, leading to PI3K/AKT activation to enhance tumour progression. Our work also provides evidence that disrupting the NLRP6/p85α interaction to inhibit the PI3K/AKT pathway might be an effective therapeutic strategy against tumours.

## Methods

### Ethical statement

Our research complies with all relevant ethical regulations. The human sample study was approved by the Ethics Committee of Third Affiliated Hospital of Soochow University (2019 Science No. 003 and 2022 Science No. 159 (M01)) and informed consent was obtained from all human participants. No participant compensation was provided. The animal experiment protocol was approved by Ethics Committee of Soochow University.

### Cell culture

Human HEK293T and U251 cell lines were purchased from National Collection of Authenticated Cell Cultures (Shanghai, China). Human LN229 cell line was purchased from Procell Life Science & Technology Co., Ltd. (Wuhan, China). Human LN18 and HS683 cell lines were purchased from Nanjing BEB Laboratories Co., Ltd. (Nanjing, China). The cultured cells were maintained in Dulbecco’s modified Eagle’s medium (Thermo Fisher Scientific, MA, USA) supplemented with 10% foetal bovine serum (Thermo Fisher Scientific, MA, USA), 100 U/mL penicillin (Thermo Fisher Scientific, MA, USA), and 100 mg/mL streptomycin (Thermo Fisher Scientific, MA, USA) at 37 °C under a humidified atmosphere with 5% CO_2_. All cell lines were recently authenticated by STR profiling and were confirmed to be free of mycoplasma by a PCR-based method. The primers for 16 S rDNA were as follows:

forward: 5′-ACTCCTACGGGAGGCAGCAGTA-3′;

reverse: 5′-TGCACCATCTGTCACTCTGTTAACCTC-3′.

The mycoplasma test was carried out every 2 weeks. All the cells were cultured for no more than 2 months to maintain a low passage number.

### Antibodies and reagents

The specific antibodies used in this study were as follows: anti-Flag (Sigma Aldrich, #A8592, 1:2000), anti-HA (Roche Applied Science, #3F10, 1:2000), anti-Myc (Santa Cruz Biotechnology, #sc-40, 1:1000), anti-GFP (Santa Cruz Biotechnology, #sc-9996, 1:1000), Streptavidin-HRP (Thermo Fisher Scientific, #434323, 1:1000), anti-NLRP6 (OriGene, #TA337214, 1:1000), anti-PTEN (Santa Cruz Biotechnology, #sc-7974, 1:1000), anti-p85α for immunohistochemistry (Santa Cruz Biotechnology, #sc-376112, 1:100), anti-p85α (Cell Signalling, #13666) for immunofluorescence (1:200), western blot (1:1000) and coimmunoprecipitation (1:1000), anti-pS473-AKT (Cell Signalling, #4060 S, 1:2000), anti-pT308-AKT (Cell Signalling, #9275 S, 1:1000), anti-AKT (Cell Signalling, #4691 S, 1:1000), anti-RBX1 (Santa Cruz Biotechnology, #sc-393640, 1:200), anti-SKP1 (Santa Cruz Biotechnology, #sc-5281, 1:200), anti-OPTN (Novus Biologicals, #NBP1-84682, 1:1000), anti-ATG5 (Cell Signalling, #12994 S, 1:1000), anti-LC3 (Cell Signalling, #3868 S, 1:1000), anti-Ubiquitin (Cell Signalling, #3936 S, 1:1000), anti-Cullin-1 (Abcam, #ab75817, 1:1000), anti-His (Cell Signalling, #9991 S, 1:1000), anti-GST (Beyotime Biotechnology, #AG768, 1:1000), anti-β-actin (Sigma Aldrich, #A2228, 1:3000; Cell Signalling, #4970, 1:3000), anti-Rabbit IgG (Beyotime Biotechnology, #A7016, 1:1000), anti-Mouse IgG (Beyotime Biotechnology, #A7028, 1:1000), anti-rabbit IgG HRP-linked Antibody (Cell Signalling, #7074 S, 1:1000), anti-mouse IgG HRP-linked antibody (Santa Cruz Biotechnology, #sc-2005, 1:1000), Goat anti-Rabbit IgG (H + L) Cross-Adsorbed Secondary Antibody Alexa Fluor 568 (Invitrogen, #A-11011, 1:500), Goat anti-Mouse IgG (H + L) Cross-Adsorbed Secondary Antibody Alexa Fluor 633 (Invitrogen, #A-21050, 1:500), Goat anti-Mouse IgG (H + L) Highly Cross-Adsorbed Secondary Antibody, Alexa Fluor 568 (Invitrogen, #A-11031, 1:500), and Goat anti-Rabbit IgG (H + L) Highly Cross-Adsorbed Secondary Antibody Alexa Fluor 488 (Invitrogen, #A-11034, 1:500). 3×Flag peptide (#P9801) was obtained from Beyotime Biotechnology (SHH, China). Cycloheximide (CHX), DAPI, NH_4_Cl, chloroquine phosphate (CQ), 3-methyladenine (3-MA), and MG132 were obtained from Sigma Aldrich (MA, USA). Bafilomycin A1 (BafA1) was obtained from Selleck Chemicals (TX, USA). MK-2206 and SC-79 were obtained from MedChemExpress (NJ, USA). Peptides Pep1-Pep11, the mutant of Pep9 (Pep9 M13), cell penetrating peptide (CPP) TAT, and TAT-linked peptides were synthesized and purified by Nanjing BEB Laboratories Co., Ltd. (Nanjing, China).

### RNA interference and quantitative real-time PCR (qRT–PCR)

The siRNAs for 22 Nod-like receptors (NLRs) were designed and synthesized by GenePharma (Shanghai, China). The siRNA targeting each NLR was transfected into cells using RNAiMAX (Thermo Fisher Scientific, MA, USA) according to the manufacturer’s instructions. The sequences of siRNA oligonucleotides for 22 NLRs are listed in Supplementary Data 1. Total RNA was extracted using TRIzol (Thermo Fisher Scientific, MA, USA) according to the manufacturer’s instructions and quantified on a Nanodrop One spectrophotometer (Thermo Fisher Scientific, MA, USA). First-strand cDNA was generated by reverse transcription of total RNA using a HiScript III 1st Strand cDNA Synthesis Kit (Vazyme, Nanjing, China). qRT–PCR was performed on an Applied Biosystems 7500 PCR (Thermo Fisher Scientific, MA, USA). The primers were designed and synthesized by GenePharma (Shanghai, China) as listed in Supplementary Data 2.

### Plasmid construction and mutagenesis

The human genes *NLRP6*, *PIK3R1*, *PTEN*, *OPTN*, *RBX1*, *SKP1*, subunits of PI3K, and other plasmids mentioned were generated by PCR amplification from a normal brain cDNA library and cloned into Flag-/HA-/Myc/GFP-tagged vectors. The truncations of NLRP6 and p85α were generated by PCR amplification and were constructed by standard subcloning. p85α mutants were generated using a site-directed mutagenesis kit (SBS Genetech, Beijing, China) according to the manufacturer’s instructions. Construction of the lenti-CRISPR/Cas9 vectors targeting the *NLRP6*, *PIK3R1*, *PTEN*, *RBX1*, *SKP1*, *CUL1*, and other lenti-CRISPR/Cas9 vectors mentioned were performed following a standard protocol. Briefly, gRNA was synthesized and annealed and then ligated into the vector at the BsmBI restriction sites. The guide RNA sequences are listed in Supplementary Data 3. The plasmid was transfected into cells with Lipofectamine 3000 reagent (Thermo Fisher Scientific, MA, USA) according to the manufacturer’s instructions.

### Generation of stable cell lines

To generate stable cell lines, lentivirus was packaged in HEK293T cells using the ViraPower Kit (Thermo Fisher Scientific, MA, USA) according to the manufacturer’s instructions. The lentivirus-containing supernatant was collected from HEK293T cells twice every 24 h during a 48 h transfection period. The glioma cell lines were infected with the lentivirus and selected by puromycin incubation. Puromycin-resistant cells were collected and cultured for further analysis. All the transfected cells were periodically tested for mycoplasma and were used within 6 weeks to minimize cell genetic drift or contamination.

### FOXO luciferase assay

PI3K/AKT pathway activity was monitored using the FOXO Reporter kit (PI3K/AKT Pathway) (BPS Bioscience, CA, USA) according to the manufacturer’s protocol^60^. Briefly, the cells were cotransfected with specific siRNA and the reporter, which was the combination of the FOXO luciferase reporter vector and Renilla luciferase vector using Lipofectamine 2000 reagent (Thermo Fisher Scientific, MA, USA). The negative control reporter (NC Reporter) served as the negative control, and the *FOXO3* vector served as the positive control. The cells were lysed and the luciferase activity was determined using the Dual-Luciferase Reporter Assay System (Promega, WI, USA).

### AKT pathway phosphorylation array

The Human AKT Pathway Phosphorylation Array kit (Raybiotech, Guangzhou, China) was used to detect the relative levels of phosphorylation of 18 AKT pathway proteins^61^. The name and position for all 18 AKT pathway proteins are listed in Supplementary Data 4. In brief, the membrane was incubated with blocking buffer at room temperature for 30 min. The blocking buffer was discarded, and the protein sample was added to the well for complete incubation at 4 °C overnight. The membrane was washed using Wash Buffer I and Wash Buffer II and incubated with Detection Antibody Cocktail at 4 °C overnight. The membrane was washed and incubated with HRP-anti-rabbit IgG at room temperature for 2 h. The membrane was then incubated with the detection buffer mixture at room temperature for 2 min. Finally, the membrane was photographed under a chemiluminescence imaging system. Data collection was performed on Array Vision Evaluation 8.0 (GE, MA, USA). The relative fold change of the protein on two different arrays was calculated according to the formula in the user manual. The differential protein was determined according to the manufacturer’s criteria: (1) mean signal density > 150; (2) fold change ≤0.83 or ≥1.2.

### Identification of NLRP6-interacting proteins

The APEX2-based proximity-tagging method combined with mass spectrometry was chosen to identify potential NLRP6-interacting proteins in live cells, the same strategy used in our previous research^62^. To construct a functional NLRP6-APEX2 fusion protein for proximity labelling, APEX2 was genetically fused to NLRP6. The NLRP6-APEX2 plasmid was transfected into cells. After 48 h, the cells were incubated with biotin phenol for 30 min at 37 °C followed by hydrogen peroxide treatment at the concentration of 2 mM for exactly 1 min. APEX2 catalysed biotin-phenol into a biotin-phenol radical that covalently bound to proteins nearby NLRP6 neighbouring (<20 nm). The cells that did not undergo biotin phenol treatment but were subjected to hydrogen peroxide treatment were served as the control. The reaction was halted by removing the media, and the cells were washed three times with quencher buffer (5 mM Trolox and 10 mM sodium ascorbate in DPBS). The cells were then lysed and the biotinylated proteins were collected by streptavidin beads. The proteins were separated by one-dimensional sodium dodecyl sulfate–polyacrylamide gel electrophoresis (SDS-PAGE) and then visualized by Coomassie blue staining. The gel pieces were excised from SDS-PAGE and were transferred to new tubes. This experiment was performed once with three replicates per condition. The proteins from three replicates in either the experimental group or the control group were blended together to create a single experimental sample and a single control sample. The sample preparation and liquid chromatography-tandem mass spectrometry (LC-MS/MS) analysis were performed by FitGene Biotechnology (Guangzhou, China). Briefly, the gel pieces were washed twice with ultrapure water and incubated in a 50% methanol/50 mM NH_4_HCO_3_ solution at 37 °C for 30 min. Dehydration was achieved by adding 100% acetonitrile. Subsequently, the dried gel pieces were incubated with 25 mM Dithiothreitol/50 mM NH_4_HCO_3_ at 56 °C for 30 min, followed by addition of 55 mM Iodoacetamide/50 mM NH_4_HCO_3_ in the dark for 30 min. The samples were then washed three times with ultrapure water and dehydrated with 100% acetonitrile. Enzymatic digestion was performed by treating the samples with trypsin at a concentration of 20 ng/µl in 25 mM NH_4_HCO_3_ at 37 °C overnight. The enzymatic hydrolysis products were purified and concentrated using ZipTip (ZTC18S096, Millipore, MA, USA). The resulting products were dissolved in 0.1% formic acid/2% acetonitrile, and the supernatants were collected for LC-MS analysis. The samples were loaded and separated on columns (#160454, #160321, Thermo Scientific, MA, USA) using the Ultimate 3000 RSLCnano system (Thermo Scientific, MA, USA). The mobile phase was consisted of 0.1% formic acid (Solvent A) and 0.1% formic acid/80% acetonitrile (Solvent B), a with a flow rate set at 300 nL/min. The initial Solvent B composition was 5% and was maintained for 5 min. It was then increased to 50% over 45 min and then increased to 90% in 5 min, and maintained at 90% for 5 min before being decreased to 5% within 10 min. The tandem mass spectrometry was performed using Q Exactive mass spectrometer (Thermo Scientific, MA, USA). The MS1 survey scan (350–1800 m/z) was conducted at a resolution of 70,000 at 200 m/z with automatic gain control (AGC) set of 3e6 and a maximum injection time of 40 ms. The most-intense 20 ions in each MS spectrum were selected for MS2 analysis at higher-energy collisional dissociation model, with an isolation window of 2 m/z. The MS2 scan was performed at a resolution of 17,500 at 200 m/z, with AGC set to 1e5, normalized collision energy of 27 eV, and a maximum injection time of 60 ms. The raw data were processed and converted using MassMatrix File Conversion Tool (Version 3.5) and searched using MASCOT engine Version 2.2 (Matrix Science, London, UK) against Uniprot Human database (uniprot_Homo_sapiens_156914_20170310.fasta) for protein identification. The following options were used: Fixed modifications = Carbamidomethylation (C), Variable modification = Oxidation (M), Enzyme = Trypsin, Maximum Missed Cleavages = 2, Peptide Mass Tolerance = 20 ppm, Fragment Mass Tolerance = 0.6 Da, Mass values = Monoisotopic, and Significance threshold = 0.05. Proteins were identified based on at least one unique peptide. The utilization of LC-MS/MS experiments to analyse and screen interacting proteins introduces the possibility of false positives, despite efforts to narrow the threshold or repeat the process. However, there is a concern about excluding genuine positive proteins due to variations in protein abundance. To address this issue, we set a slightly wider threshold to minimize such exclusions. Though we couldn’t entirely eliminate nonspecific and contaminant proteins, we successfully identified a significant group of target proteins. Subsequently, Co-IP analysis was conducted to confirm the interactions with NLRP6, and these findings were further validated through other various independent experiments. As NLRP6 is involved in PI3K/AKT pathway regulation, p55γ was first selected from the list. However, p55γ did not interact with NLRP6 in our preliminary experiment. Because the peptide sequence was also the same as part of the p85α peptide sequence, p85α was investigated. To detect ubiquitinated p85α, a Flag-tagged p85α plasmid was transfected into cells. The cell lysates were immunoprecipitated with an anti-Flag antibody and separated on a 8% SDS–PAGE gel. The experiment was performed once with three replicates. The proteins from three replicates were blended together to create one sample for further analysis. The sample preparation and LC-MS/MS analysis was performed by Applied Protein Technology (Shanghai, China). Briefly, after in gel digestion which was the same as described above, the resulting sample was preceded for LC-MS/MS analysis. The sample was loaded onto C18 trap column (Thermo Scientific Acclaim PepMap100, 2 cm, 100 μm) and was separated by C18 analytical column (Thermo Scientific Easy Column, 10 cm, 75 μm, 3 μm resin) on the EASY-nLC1000 system (Thermo Scientific, MA, USA). The flow rate was controlled at 300 nL/min. The mobile phase was consisted of 0.1% formic acid (Solvent A) and 0.1% formic acid/80% acetonitrile (Solvent B). The Solvent B increased from 0 to 35% during 50 min, from 35–100% during from 50–55 min, and maintained at 100% from 55–60 min. The tandem mass spectrometry was performed by Q Exactive mass spectrometer (Thermo Scientific, MA, USA). The mass spectrometer was operated in positive ion mode. The MS1 survey scan (300–1800 m/z) was at a resolution of 70,000 at 200 m/z with automatic gain control (AGC) target of 3e6 and a maximum injection time of 10 ms. Dynamic exclusion was 40.0 s. The most-intense 20 ions in each MS spectrum were selected for MS2 analysis at higher-energy collisional dissociation model, with an isolation window of 2 m/z. MS2 scan was at a resolution of 17,500 at 200 m/z with normalized collision energy 30 eV. The underfill ratio was defined as 0.1%. The MS/MS spectra were searched Proteome Discoverer 1.4 (Thermo Electron, CA, USA) against Uniprot Human database (Swissprot_human_20368_20200217.fasta). The following options were used: Enzyme = Trypsin, Max Missed cleavages = 2, Fixed modifications = Carbamidomethyl (C), Variable modification = Oxidation(M), GlyGly(K), Max Missed Cleavages = 2, and Filter by Peptide Confidence = High. The score was calculated as −10 × Log_10_ (P), where P is the absolute probability. The Exponentially Modified Protein Abundance Index (emPAI), a measure of protein abundance in a single LC-MS/MS experiment, was utilized for the analysis of coimmunoprecipitation interacting proteins^63^. Proteins were identified based on the presence of at least one unique peptide. To ensure high confidence in the protein and peptide identification, a threshold of 1% False Discovery Rate (FDR) was established using the decoy database approach. Additionally, the tandem mass spectra of matched ubiquitinated peptides underwent manual validation to ensure their accuracy and reliability. The list of NLRP6-interacting proteins by LC-MS/MS analysis is shown in Supplementary Data 5. Data are available via ProteomeXchange with identifier PXD037460.

### Coimmunoprecipitation, ubiquitination assay, and immunoblotting

For coimmunoprecipitation, cell lysate was collected and incubated with the indicated antibody at 4 °C overnight followed by incubation with Protein A/G beads (Pierce, IL, USA) at 4 °C for 2 h. The unbound proteins were washed away with lysis buffer. The precipitated proteins were separated from the beads. To detect protein expression in frozen tissues or cells, total protein was extracted using RIPA lysis buffer (Beyotime, Shanghai, China) supplemented with protease and phosphatase inhibitors (Beyotime, Shanghai, China). The protein concentration was determined using a BCA protein quantification kit (Beyotime, Shanghai, China) or BCA Protein Assay Kit (BEB, Nanjing, China). The proteins were separated by SDS–PAGE, transferred to polyvinylidene fluoride (PVDF) membranes (Bio–Rad, CA, USA), and incubated with the indicated antibodies. The bands were detected with the ECL Advanced Western blot Detection Kit (Thermo Fisher Scientific, MA, USA) or Super ECL Chemiluminescent Substrate Kit (BEB, Nanjing, China) on a ChemiDoc Touch System (Bio–Rad, CA, USA) or Tanon 4600 (Tanon, Shanghai, China). The band intensities were quantified using Image Lab software (Bio–Rad, CA, USA) or ImageJ software (National Institutes of Health, MD, USA).

For the ubiquitination assay, the cell lysates were prepared by using low-salt buffer containing protease inhibitors (Roche, Mannheim, Germany) in the presence of 1% SDS. The extracts were boiled at 95 °C for 5–10 min to denature the samples and the samples were diluted by low-salt buffer containing protease inhibitors to 0.1% SDS for coimmunoprecipitation. Finally, the immunoprecipitates were collected for the following immunoblot analysis experiments.

For the denaturation coimmunoprecipitation experiment, the cells were washed twice with PBS and lysed in a low salt buffer containing 150 mM NaCl, 20 mM Tris-HCl (pH 7.4), 0.5 mM EDTA, 1% Nonidet P-40 (NP-40) and protease inhibitors (Roche, #11836170001). The cell lysates were centrifuged at 12,000 g at 4 °C for 20 min, and the protein concentration was measured using a BCA assay kit (Thermo Fisher Scientific, #23225). Equal amounts of lysates were used for either whole cell lysates immunoblotting or subsequent immunoprecipitation. The lysates were denatured by SDS buffer to a 1% SDS final concentration and then diluted 20-fold in low salt buffer to reduce the SDS concentration to ≤0.1%. Next, Flag-beads (Thermo Fisher Scientific, #A36804) were added to the samples and incubated on a rotor at 4 °C overnight. Then, the equivalent amount of cell lysates were added into the samples and rotated at 4 °C overnight. After washing five times with low salt buffer, the immunoprecipitates were eluted with 2 × SDS sample buffer and boiled at 95 °C for 10 min. Samples were then immediately loaded on 4–20% polyacrylamide gels and analysed by immunoblot analysis.

### Recombinant protein purification

DNA encoding full-length NLRP6 was codon optimized and chemically synthesized by Genewiz (Soochow, China). The DNA sequence was subcloned into NdeI-XhoI sites of a pDB-His-MBP vector (kindly given by Pro. Chenggui Han, China Agricultural University, Beijing, China). Recombinant NLRP6 protein was expressed and purified following previous reports, with slight modifications^64^. Briefly, the protein was expressed in *E. coli* (DE3) at 16 °C with 0.5 mM IPTG induction overnight. Then, the protein was purified by Ni affinity and gel filtration chromatography. The p85α WT and p85α mutant sequences were chemically synthesized and subcloned into BamHI-XhoI sites of a pGEX-6P-1 plasmid by GenScript (Nanjing, China). The *E. coli* (DE3) transformed with the plasmids were grown in LB medium at 37 °C. The culture medium was supplemented with 0.5 mM IPTG followed by incubation at 16 °C for 16–18 h. The proteins were purified by glutathione-affinity chromatography (Smart-Life Sciences, Changzhou, China) followed by gel filtration chromatography (Cytiva, MA, USA) using PBS. The protein extinction coefficient was calculated by ProtParam, and the protein concentration was determined by the ultraviolet spectrophotometry method^65^.

### Immunofluorescence

The cells seeded on glass bottom culture dishes (Nest Scientific, NJ, USA) were fixed with 4% paraformaldehyde in PBS pH 7.4 for 15 min at room temperature. The cells were incubated in 100% methanol for 10 min at −20 °C. The cells were then washed three times with ice-cold PBS, and they were incubated with 5% foetal goat serum for 1 h to block nonspecific binding and then with diluted primary antibodies in a humidified chamber overnight at 4 °C. The cells were washed three times in PBS and incubated with fluorescently labelled secondary antibodies for 1 h at room temperature in the dark. The cells were washed three times in PBS in the dark. The cells were finally imaged under Leica TCS-SP8 confocal microscope equipped with a ×100 NA oil-immerson objective (Leica, Mannheim, Germany). The images were analysed using ImageJ software (National Institutes of Health, MD, USA).

### Cell growth assay

Cells were seeded into 12-well plates at a density of 1 × 10^4^ cells per well. Manual cell counting was carried out at the indicated times using trypan blue and a haemocytometer.

### Colony formation assay

Cells were collected and seeded in 6-well plates at the density of 500 or 1000 cells per well in DMEM containing 10% FBS for 2-3 weeks. The plates were stained with crystal violet (Beyotime Biotechnology, Shanghai, China) and were counted using ImageJ software (National Institutes of Health, USA).

### Wound healing assay

Cells were seeded in 6-well plates and grown to 70% confluence. The cell monolayers were scraped with a 10 µl pipette. Wound closure was monitored at the indicated times, and images were taken under a microscope (IX71, Olympus, Japan).

### Animals and intracranial xenograft

BALB/c nude mice were maintained and bred in a specific-pathogen free (SPF) environment, adhering to standard conditions of temperature (20–26 °C) and humidity (40–70%). They were subjected to a strict 12-h light cycle, with lights on at 08:00 a.m. and off at 08:00 p.m. No limitations were imposed on the sex of the experimental animals involved in this study. Cells expressing firefly luciferase mixed with Matrigel (Corning, NY, USA) were intracranially injected into 5- to 6-week-old female athymic nude mice. The intracranial injection point was at the cerebral cortex, 1 mm prior to coronal suture, 1 mm on the right side of the centreline, and 3 mm below the dura mater. Appropriate medications were provided to reduce pain. To monitor intracranial tumour growth, the animals were intraperitoneally injected with D-luciferin (Yeasen, #40902ES01) and anaesthetized with isoflurane. The images were captured using an In Vivo MS FX pro Imaging System (Bruker, MA, USA) or IVIS Lumina imaging station (Perkin Elmer, MA, USA). The results were reported as the total flux (photons/second). The mice were sacrificed at the indicated time points. The brains were removed, fixed in 4% paraformaldehyde, and embedded in paraffin.

### Clinical samples

The pathologic diagnosis was performed by two independent pathologists. Fifty-seven GBM samples obtained between January 2008 and March 2022 were determined to be PTEN wild type by PCR single-strand conformation polymorphism (PCR-SSCP) according to previous methods^66^. Briefly, genomic DNA was extracted from approximately 25 mg tumour samples using QIAamp DNA Mini Kit (Qiagen, CA, USA) according to the manufacturer’s instructions and quantified on a Nanodrop One spectrophotometer (Thermo Fisher Scientific, MA, USA). The primers for different *PTEN* exons are listed in Supplementary Data 6. The PCR reactions were performed on an Applied Biosystems 7500 PCR (Thermo Fisher Scientific, MA, USA) and Veriti Thermal Cycler (Thermo Fisher Scientific, MA, USA). The PCR product was denatured and electrophoresed in an 8% polyacrylamide gel for 12–18 h at 14 °C. Silver staining was performed to visualize DNA bands. The demographic and clinical features of these samples are listed in Supplementary Table 1.

### Immunohistochemistry and scoring

In brief, the tissue sections were deparaffinized in xylene and 50–100% ethanol sequentially and then rehydrated in deionized water. The sections were immersed in Antigen Retrieval Solution in a pressure cooker and kept at 120 °C for 2.5 min. Endogenous peroxidase activity was blocked with peroxidase blocking reagent (3% hydrogen peroxide in water) for 15 min. The sections were incubated with primary antibodies in incubation buffer at 4 °C overnight. The sections were rinsed with wash buffer and incubated with secondary antibodies at room temperature for 1 h. Finally, the sections were immersed in DAB Chromogen Solution for suitable staining and counterstained with Haematoxylin QS for 3 min. The protein score was calculated by multiplying the percentage of positive cells (<10% = 0, 10–25% = 1, 25–50% = 2, 50–75% = 3, >75% = 4) by the intensity (negative = 0, weak staining = 1, moderate staining = 2, or strong staining = 3). The stained sections were evaluated by two independent pathologists who were blinded to the clinical characteristics.

### Biolayer interferometry (BLI) assay

Eleven α-helical peptides were synthesized (Supplementary Data 7). Purified recombinant NLRP6 was biotinylated in assay buffer (PBS with 0.01% Tween-20) at room temperature for 1 h. The interaction between the peptides and NLRP6 was determined by BLI using an Octet Red 96 instrument (FortéBio, CA, USA). Loading of streptavidin biosensors was conducted by exposing pre-equilibrated biosensor tips in PCR tubes containing 15 μl biotin-NLRP6 (50 μg/ml) for 1 h at room temperature. All of the binding data were collected at 30 °C. The experiment was composed of three steps: (1) baseline, (2) association, and (3) dissociation. Responses (nanometre shift) were calculated using data that were double reference subtracted using reference wells and nonspecific binding of the biosensor to the analyte. Global 1:1 fitting of association and dissociation curves with FortéBio data analysis 9.0 software revealed k_on_, k_dis_, and K_D_ binding constants. GraphPad Prism 9.0 was used to visualize curves.

### ELISA

The 96-well plate was coated with bovine serum albumin (BSA) or purified NLRP6. HRP-conjugated PepC or HRP-conjugated Pep9 was incubated with BSA or NLRP6 for 1 h, and the unbound peptide was washed away. The HRP substrate was added, and the absorbance was detected at 450 nm. For the competitive ELISA, the 96-well plate was coated with purified p85α. The binding of NLRP6 to p85α competed with 10 μM PepC or 5 μM, 10 μM, 20 μM Pep9 for 1 h.

### Homology modelling and docking

The 3D structure of the human p85α RHO domain was extracted from the Protein Data Bank (PDB) with PDB code of 1PBW [https://www.rcsb.org/structure/1PBW]. The structure of the NLRP6 NOD domain was built using I-TASSER^31^. The complex structure of p85α with NLRP6 was generated using the protein–protein docking tool CoDockPP^32^. The structure of the NLRP6 NOD domain was set as the receptor, and the structure of the p85α RHO domain was set as the ligand. The angle step was set at 15°, and the cut-off value was set at 2 Å. The top 10 poses were retained according to the knowledge-based scoring function.

### Peptide treatment

To determine the antitumour effect of the peptide in vitro, the peptide was incubated with cells for 24 h when the cells were grown to approximately 80% confluence. Cell viability was measured at the indicated time points. The cell lysates were collected for immunoprecipitation as described above. To determine the antitumour effect of the peptide in vivo, the peptide was stereotactically injected using identical coordinates of tumour implantation in 3 μl of PBS. Briefly, 3 weeks after intracranial tumour cell implantation, mice with similar tumour sizes and similar weights were selected and randomly arranged into five groups (*n* = 6): PBS, PepC (0.25 mg/kg TAT-PepC per injection), Pep9 low dose (Pep9 L, 0.05 mg/kg TAT-Pep9 per injection), Pep9 medium dose (Pep9 M, 0.1 mg/kg TAT-Pep9 per injection), and Pep9 high dose (Pep9 H, 0.25 mg/kg TAT-Pep9 per injection). Each mouse received intratumoural injection every 10 days (at Day 21, Day 31, Day 41, and Day 51), and tumour growth was monitored at Day 21, Day 41, and Day 56. The experiment was terminated at Day 80, and all the mice (if they were alive) were sacrificed as previously described.

### Statistical analysis

The Student’s *t* test or survival analysis was performed using GraphPad Prism 9.0. The heatmap was created by TBtools^67^. The protein domain structure was created by Illustrator for Biological Sequences 2.0^68^. *p* values less than 0.05 were considered statistically significant.

### Supplementary information

Supplementary Information

Description of Additional Supplementary Files

Supplementary Data 1

Supplementary Data 2

Supplementary Data 3

Supplementary Data 4

Supplementary Data 5

Supplementary Data 6

Supplementary Data 7

nr-reporting-summary

### Source data

Source Data

## Data Availability

The mass spectrometry proteomics data have been deposited to the ProteomeXchange Consortium via the PRIDE partner repository with the dataset identifier PXD037460. The 3D structure of the human p85α RHO domain is extracted from the Protein Data Bank archive 1PBW. All data supporting the findings of this study are included in the manuscript and its supplementary files are available. Source data are provided with this paper.
