# Supplementary material for: NLRP6 potentiates PI3K/AKT signalling by promoting autophagic degradation of p85α to drive tumorigenesis
Source: Nat Commun. 2023 Sep 28;14:6069. doi: 10.1038/s41467-023-41739-z (PMC10539329; doi:10.1038/s41467-023-41739-z)
Supplement: Supplementary file 1 — Supplementary Information [file 41467_2023_41739_MOESM1_ESM.pdf]

NLRP6 potentiates PI3K/AKT signalling by promoting autophagic degradation of p85α to drive tumorigenesis

Zhi *et al.*

Inventory of Supporting Information

1. Supplementary Figures and figure legends

Supplementary Figure 1. Identification of NLRP6 as a positive regulator of the PI3K/AKT pathway.

Supplementary Figure 2. NLRP6 regulates the PI3K/AKT pathway through PTEN.

Supplementary Figure 3. NLRP6 interacts with p85α and promotes p85α protein degradation.

Supplementary Figure 4. NLRP6 promotes p85α ubiquitination at K256.

Supplementary Figure 5. NLRP6 recruits RBX1 to ubiquitinate p85α.

Supplementary Figure 6. NLRP6 promotes glioma tumorigenesis.

Supplementary Figure 7. Kinetic interactions between different peptides and recombinant NLRP6.

Supplementary Figure 8. Pep9 inhibits glioma proliferation *in vitro* and *in vivo*.

2. Supplementary Table 1. Clinical characteristics of GBM patients.

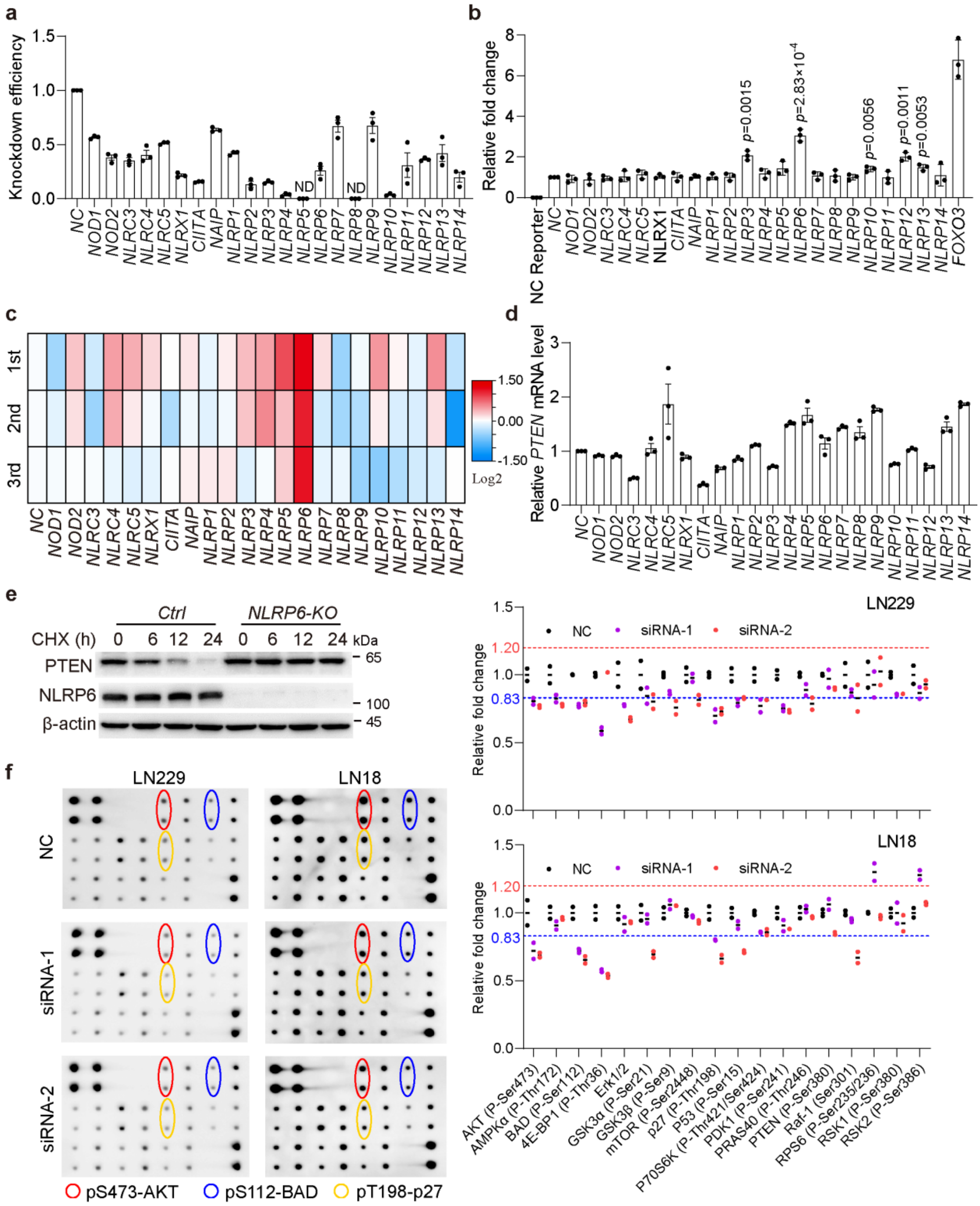

Supplementary Figure 1. Identification of NLRP6 as a positive regulator of the PI3K/AKT pathway. (a) Knockdown efficiencies of siRNAs targeting the indicated NOD-like receptors (NLRs) in LN229 cells. ND, not detected. (b) The effects of knockdown of different NLRs on FOXO transcription activity. LN229 cells were transfected the negative control (NC) Reporter, FOXO3 reporter, and indicated siRNAs. Cells were harvested, and reporter activity was measured by luciferase assay.

(c) Heatmap of relative PTEN protein expression in LN229 cells after transfection with indicated siRNAs in three independent experiments. Data were normalized to the basal protein levels and log2 transformed. (d) qRT-PCR analysis of relative *PTEN* mRNA expression in LN229 cells after transfection with the indicated siRNAs. (e) Immunoblot analysis of PTEN in control (*Ctrl*) or *NLRP6* knockout (KO) LN229 cells treated with cycloheximide (CHX, 100 μg/ml). Data shown are representative of three independent experiments with similar results. (f) Phosphorylation array of AKT pathway for *NLRP6* knockdown by siRNAs in LN229 and LN18 cells. Circles indicate proteins with significant changes in both cell lines (left). Statistical analysis of relative fold change of 18 phosphorylated proteins listed once with duplicate on the AKT Pathway Phosphorylation Array in LN229 cells and LN18 cells after transfection with NC or *NLRP6* siRNAs. The differential protein was determined by average fold change of the protein ⩽ 0.83 or ⩾ 1.2 (right). In a, b, and d, all error bars, mean values ± SEM, p-values were determined by unpaired two-tailed Student's t test of n = 3 independent biological experiments. For e, data shown are representative of three independent experiments with similar results. Source data are provided as a Source Data file.

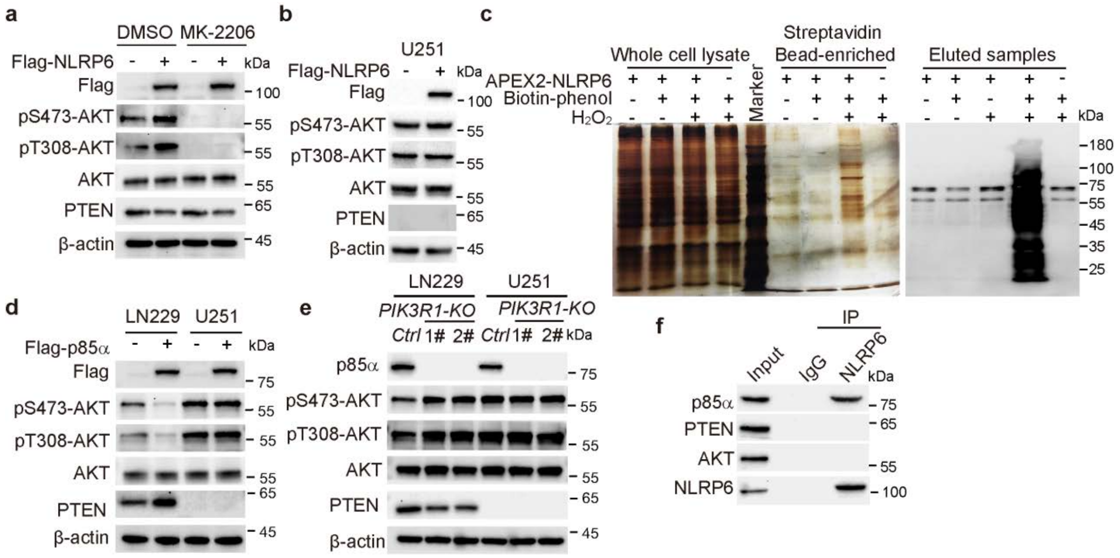

Supplementary Figure 2. NLRP6 regulates the PI3K/AKT pathway through PTEN. (a) MK-2206 inhibited PI3K/AKT pathway activation induced by NLRP6 overexpression. (b) U251 cells were transfected with an empty vector or Flag-NLRP6 vector and immunoblotted with indicated antibodies. (c) Silver staining and immunoblotting of biotinylated binding candidates near NLRP6 in LN229 cells. (d) LN229 or U251 cells were transfected with an empty vector or Flag-p85α vector and immunoblotted with indicated antibodies. (e) Immunoblot analysis of indicated proteins in Control (*Ctrl*) or *PIK3R1* knockout (KO) LN229 or U251 cells. (f) Coimmunoprecipitation (Co-IP) analysis of the interaction between NLRP6 and endogenous p85α, PTEN, or AKT in LN18 cell lysates. Data are representative of three independent experiments with similar results (f), or two independent experiments (a-b, and d-e). Source data are provided as a Source Data file.

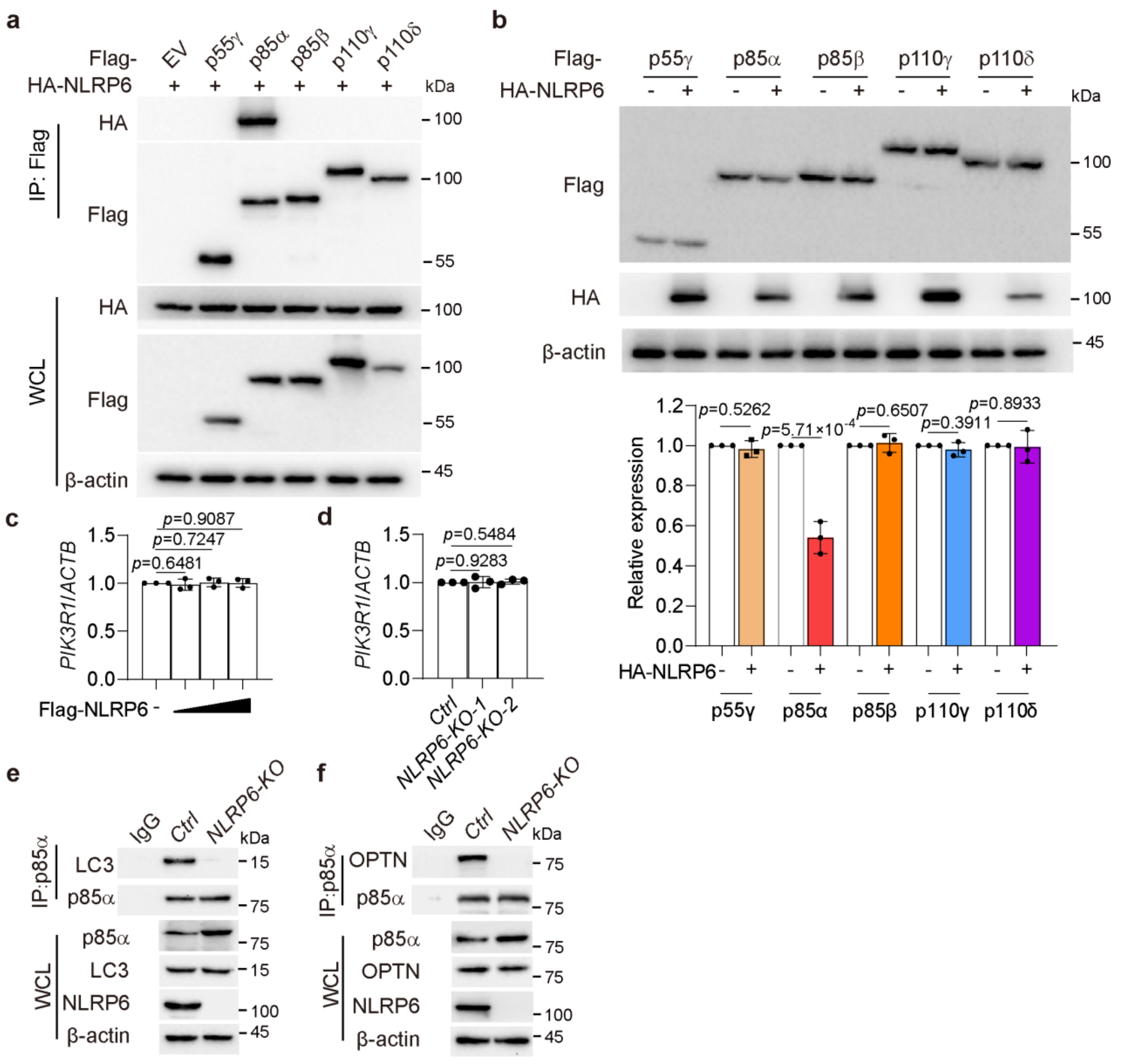

Supplementary Figure 3. NLRP6 interacts with p85α and promotes p85α protein degradation. (a) HA-NLRP6 and different Flag-tagged plasmids were cotransfected into HEK293T cells. The interaction between NLRP6 and subunits of PI3K was examined by coimmunoprecipitation (Co-IP). WCL, whole cell lysates. (b) The effect of NLRP6 overexpression on subunits of PI3K protein expression. HA-NLRP6 and different Flag-tagged plasmids were cotransfected into HEK293T cells and probed with the indicated antibodies (top). Quantification of indicated protein levels (bottom). (c) Quantification of relative *PIK3R1* mRNA levels in LN229 cells transfected with increasing amounts of Flag-NLRP6. (d) Quantification of relative *PIK3R1* mRNA

levels in *NLRP6* knockout (KO) LN229 cells. (e) Co-IP analysis of the interaction between endogenous p85α and LC3 in the absence of NLRP6 in LN18 cell lysates. (f) Co-IP analysis of the interaction between p85α and OPTN in the absence of NLRP6 in LN18 cell lysates. For a, e, and f, data shown are representative of three independent experiments with similar results. In b, all error bars, mean values ± SD, p-values were determined by unpaired two-tailed Student's t test of $n = 3$ independent biological experiments. Source data are provided as a Source Data file.

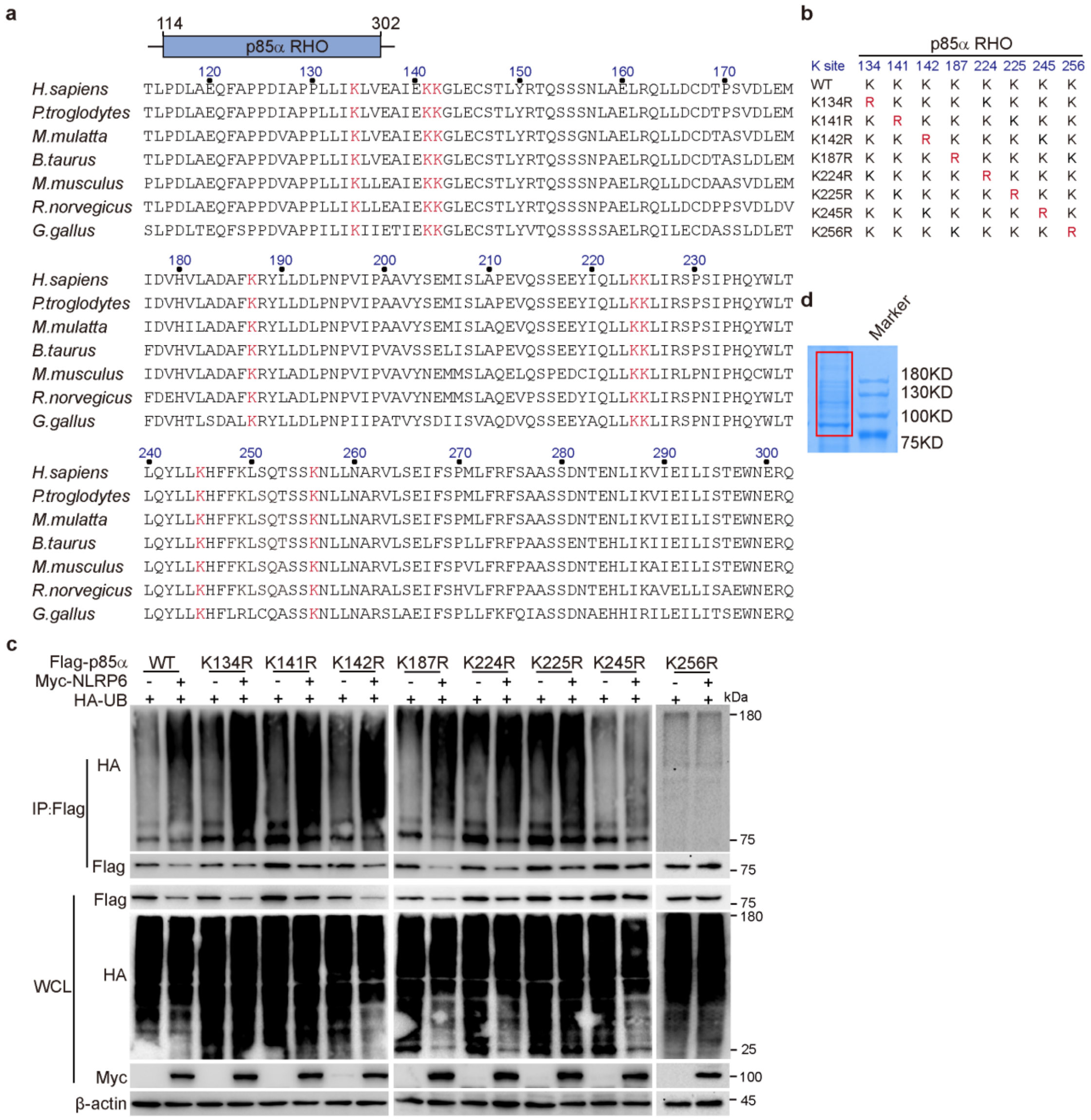

Supplementary Figure 4. NLRP6 promotes p85α ubiquitination at K256. (a) Alignment of p85α RHO domain amino acid sequences. The conserved lysine (K) on p85α is highlighted. (b) Construction of different p85α RHO domain mutants. (c) Coimmunoprecipitation (Co-IP) and immunoblot analysis of extracts of HEK293T cells transfected with Myc-NLRP6 and indicated p85α mutants along with HA-ubiquitin (UB). (d) SDS-PAGE of Flag-p85α, which was separated from LN229 cell lysates cotransfected with Flag-p85α and Myc-NLRP6. For c, data shown are representative of three independent experiments with similar results. Source data are

provided as a Source Data file.

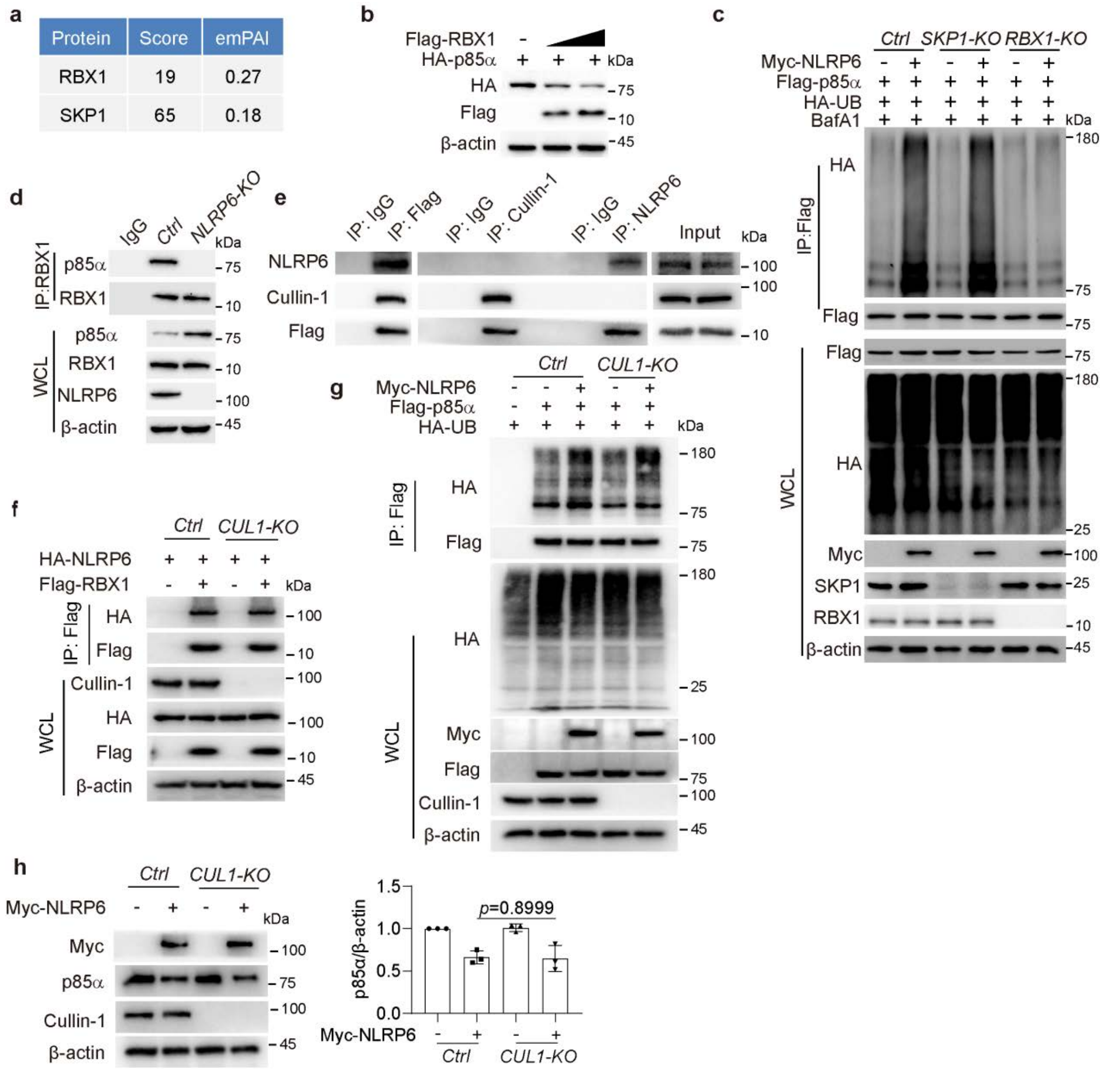

Supplementary Figure 5. NLRP6 recruits RBX1 to ubiquitinate p85α. (a) RBX1 and SKP1 were potential candidates surrounding NLRP6. (b) Immunoblot analysis of exogenous p85α in LN229 cells in the presence of increasing amounts of RBX1. (c) Coimmunoprecipitation (Co-IP) and immunoblot analysis of extracts from Control (*Ctrl*), *SKP1* knockout (KO), or *RBX1* KO LN229 cells transfected with various combinations of Myc-NLRP6, Flag-p85α, and HA-ubiquitin (UB) in the presence of bafilomycin A1 (BafA1, 0.2 μM). WCL, whole cell lysates. (d) Co-IP analysis of the interaction between p85α and RBX1 in the absence of NLRP6 in LN18 cell lysates. (e) Flag-RBX1 was transfected into LN229 cells, and its interaction with endogenous

NLRP6 or Cullin-1 was validated by Co-IP. The interaction between endogenous NLRP6 and endogenous Cullin-1 was also determined by Co-IP. (f) Flag-RBX1 and/or HA-NLRP6 were transfected into *Ctrl* or *CUL-1* KO LN229 cells and their interaction was determined by Co-IP. (g) Myc-NLRP6, Flag-p85α, and HA-UB were cotransfected into *Ctrl* or *CUL-1* KO LN229 cells in the presence of bafilomycin A1 (BafA1, 0.2 μM). Co-IP and immunoblot analysis of extracts from *Ctrl* or *CUL-1* KO LN229 cells. (h) Immunoblot analysis of extracts from *Ctrl* and *CUL-1* KO LN229 cells after transfected with Myc-NLRP6 and probing with indicated antibodies (left). Quantification of the protein levels of p85α (right). In h, all error bars, mean values ± SD, p-values were determined by unpaired two-tailed Student's t test of n = 3 independent biological experiments. Data are representative of three independent experiments with similar results (c-f), or two independent experiments (b and g). Source data are provided as a Source Data file.

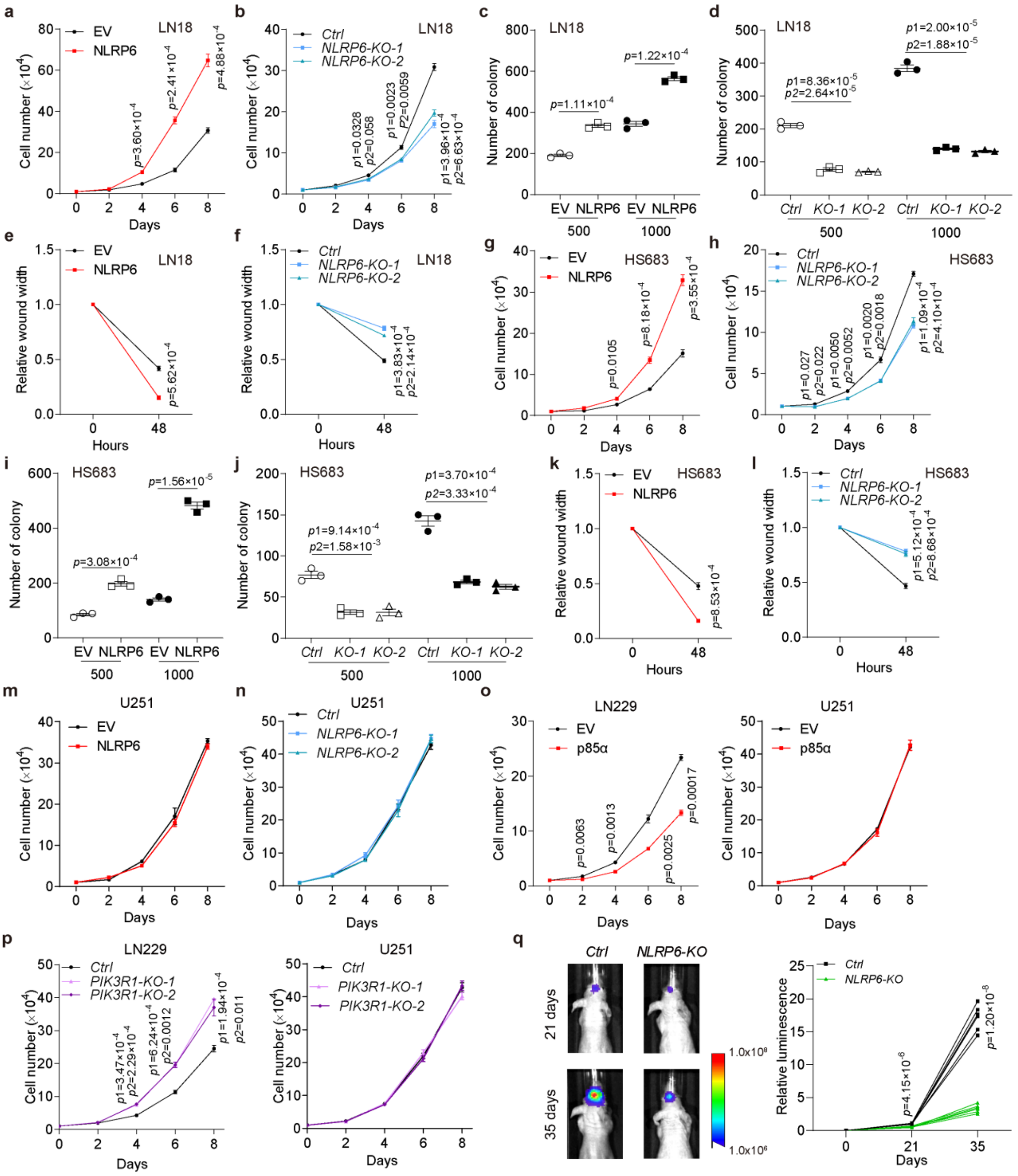

Supplementary Figure 6. NLRP6 promotes glioma tumorigenesis. (a) Cell proliferation assay of NLRP6-overexpressing LN18 cells compared with empty vector (EV)-transfected cells. (b) Cell proliferation assay of *NLRP6* knockout (KO) LN18 cells compared with control cells (*Ctrl*). (c) Colony formation assay of NLRP6-overexpressing LN18 cells compared with EV. (d) Colony formation assay of *NLRP6* KO LN18 cells compared with *Ctrl*. (e) Cell wound healing assay of NLRP6-overexpressing LN18 cells compared with EV. (f) Cell wound healing assay

of *NLRP6* KO LN18 cells compared with *Ctrl*. (g) Cell proliferation assay of NLRP6-overexpressing HS683 cells compared with EV. (h) Cell proliferation assay of *NLRP6* KO HS683 cells compared with *Ctrl*. (i) Colony formation assay of NLRP6-overexpressing HS683 cells compared with EV. (j) Colony formation assay of *NLRP6* KO HS683 cells compared with *Ctrl*. (k) Cell wound healing assay of NLRP6-overexpressing HS683 cells compared with EV. (l) Cell wound healing assay of *NLRP6* KO HS683 cells compared with *Ctrl*. (m) Cell proliferation assay of NLRP6-overexpressing U251 cells compared with EV. (n) Cell proliferation assay of *NLRP6* KO U251 cells compared with *Ctrl*. (o) Cell proliferation assay of p85α-overexpressing LN229 cells (left) and U251 cells (right) compared with controls. (p) Cell proliferation assay of *PIK3R1* KO LN229 cells (left) and U251 cells (right) compared with controls. (q) *In vivo* bioluminescence imaging of nude mice with intracranially implanted *Ctrl* or *NLRP6* KO LN229 cells. Representative bioluminescence images (left) and quantitative analysis of relative bioluminescence are shown (right). *p*1 or *p*2 was the p-value for comparing *NLRP6-KO-1* or *NLRP6-KO-2* with *Ctrl*. In a-p, all error bars, mean values ± SEM, p-values were determined by unpaired two-tailed Student's t test of n = 3 independent biological experiments. In q data show values ± SD, unpaired two-tailed Student's t test (n = 6 mice per group). Source data are provided as a Source Data file.

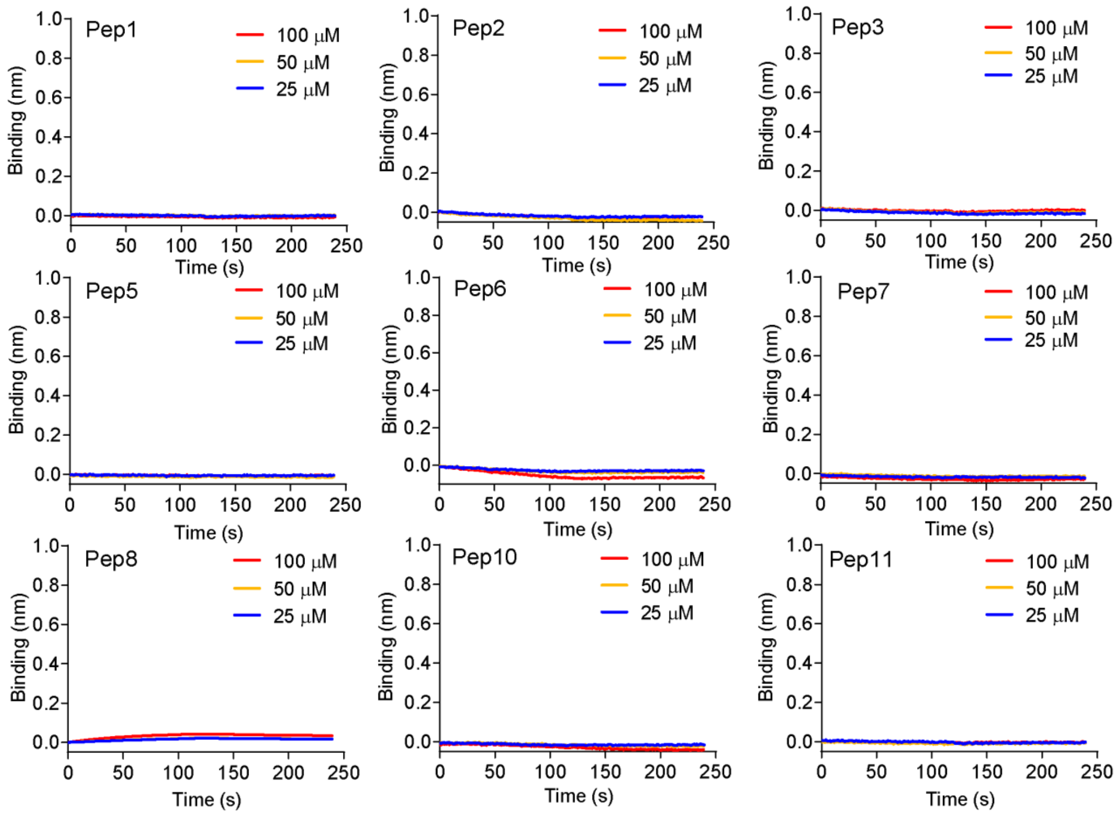

Supplementary Figure 7. Kinetic interactions between different peptides and recombinant NLRP6. Representative biolayer interferometry curves show association and dissociation kinetics for each indicated peptide with NLRP6. The buffer background was subtracted to correct curves. Three different concentrations were used for each experiment. The fitted curves obtained from global fitting analysis are displayed as dotted lines. Source data are provided as a Source Data file.

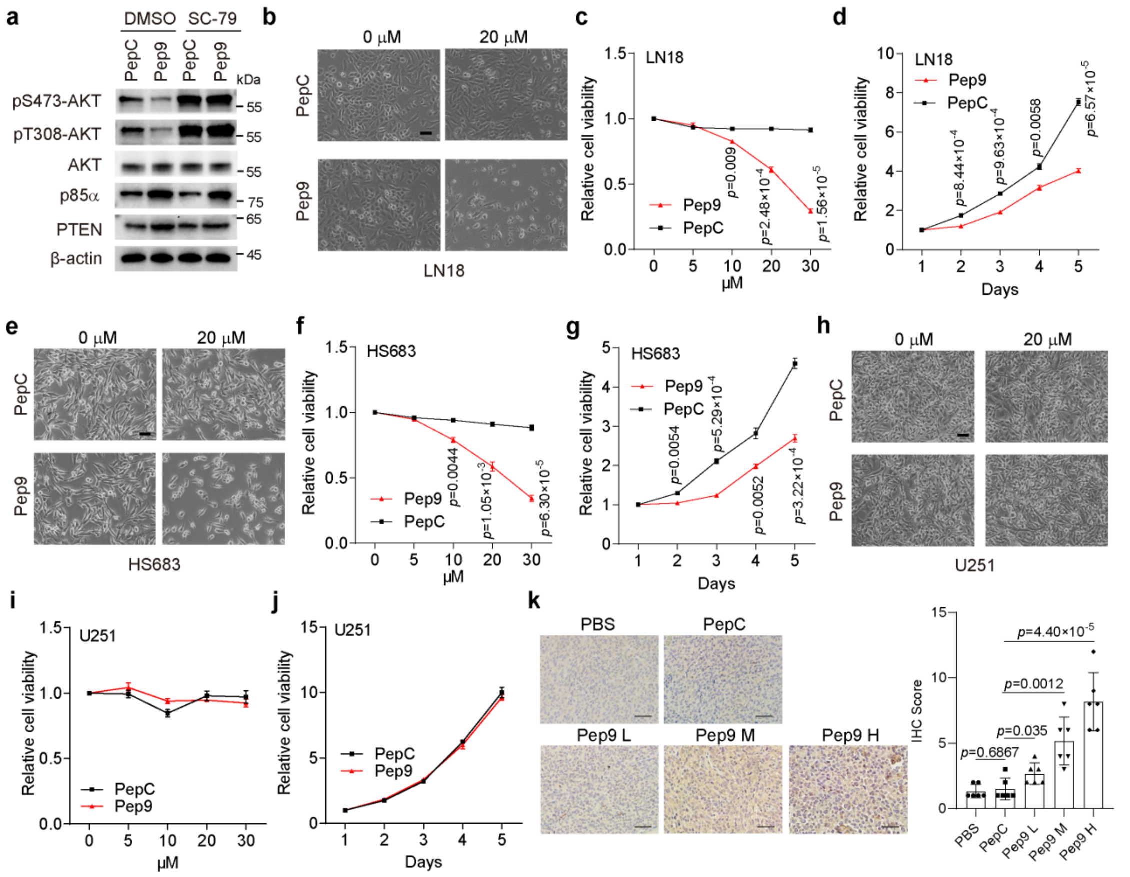

Supplementary Figure 8. Pep9 inhibits glioma proliferation *in vitro* and *in vivo*. (a) SC-79 relieved PI3K/AKT inhibition induced by Pep9. (b) LN18 cells were treated with TAT-PepC or TAT-Pep9 for 24 h. Scale bar = 50 µm. (c) Cell proliferation assay of LN18 cells treated with different concentrations of TAT-Pep9. (d) Cell proliferation assay of LN18 cells treated with TAT-Pep9 at the indicated time points. (e) HS683 cells were treated with TAT-PepC or TAT-Pep9 for 24 h. Scale bar = 50 µm. (f) Cell proliferation assay of HS683 cells treated with different concentrations of TAT-Pep9. (g) Cell proliferation assay of HS683 cells treated with TAT-Pep9 at the indicated time points. (h) U251 cells were treated with TAT-PepC or TAT-Pep9 for 24 h. Scale bar = 50 µm. (i) Cell proliferation assay of U251 cells treated with different concentrations of TAT-Pep9. (j) Cell proliferation assay of U251 cells treated with TAT-Pep9 at the indicated time points. (k) The effect of the peptide on the *in vivo* stability of p85α.

Representative images of IHC staining of p85α in tumor tissues from mice after different treatments (left). Pep9 L, Pep9 low dose, Pep9 M, Pep9 medium dose, Pep9 H, Pep9 high dose. Scale bar = 50 μm. Statistical analysis of p85α IHC score (right). In c, d, f, g, i, and j, all error bars, mean values ± SEM of n = 3 independent biological experiments. For a, b, e, and h, data shown are representative of three independent experiments with similar results. For k, all error bars, mean values ± SD, p-values were determined by unpaired two-tailed Student's t test (n = 6 mice per group). Source data are provided as a Source Data file.

Supplementary Table 1. Clinical characteristics of GBM patients.

**Variable**	Glioblastoma (n=57)	
	No.	%
**Age (years)**		
⩽ 56	31	54.3
> 56	26	45.7
**Gender**		
Male	28	49.1
Female	29	50.9
**Extent of surgery**		
gross total resection	12	21.1
< gross total resection	45	78.9
