# Supplementary material for: NLRP6 potentiates PI3K/AKT signalling by promoting autophagic degradation of p85α to drive tumorigenesis
Source: Nat Commun. 2023 Sep 28;14:6069. doi: 10.1038/s41467-023-41739-z (PMC10539329; doi:10.1038/s41467-023-41739-z)
Supplement: Supplementary file 2 — Description of Additional Supplementary Files [file 41467_2023_41739_MOESM2_ESM.docx]

**Description of Additional Supplementary Files**

File Name: Supplementary Data 1

Description: The sequences of siRNA oligonucleotides for 22 NLRs

File Name: Supplementary Data 2

Description: The sequences of the primers for the indicated genes

File Name: Supplementary Data 3

Description: The gRNA sequences of the indicated genes

File Name: Supplementary Data 4

Description: The array map of AKT pathway phosphorylation array

File Name: Supplementary Data 5

Description: Identification of NLRP6-interacting proteins by LC-MS/MS analysis

File Name: Supplementary Data 6

Description: The sequences of the primers for *PTEN* gene

File Name: Supplementary Data 7

Description: Synthesized peptide sequences, molecular weight, and purity
