# Supplementary material for: NLRP6 potentiates PI3K/AKT signalling by promoting autophagic degradation of p85α to drive tumorigenesis
Source: Nat Commun. 2023 Sep 28;14:6069. doi: 10.1038/s41467-023-41739-z (PMC10539329; doi:10.1038/s41467-023-41739-z)
Supplement: Supplementary file 10 — nr-reporting-summary [file 41467_2023_41739_MOESM10_ESM.pdf]

nature portfolio

Corresponding author(s): Jun Cui

Last updated by author(s): Sep 22, 2023

Reporting Summary

Nature Portfolio wishes to improve the reproducibility of the work that we publish. This form provides structure for consistency and transparency in reporting. For further information on Nature Portfolio policies, see our Editorial Policies and the Editorial Policy Checklist.

Statistics

For all statistical analyses, confirm that the following items are present in the figure legend, table legend, main text, or Methods section.

n/a	Confirmed	
☐	☒	The exact sample size (*n*) for each experimental group/condition, given as a discrete number and unit of measurement
☐	☒	A statement on whether measurements were taken from distinct samples or whether the same sample was measured repeatedly
☐	☒	The statistical test(s) used AND whether they are one- or two-sided<br>*Only common tests should be described solely by name; describe more complex techniques in the Methods section.*
☒	☐	A description of all covariates tested
☒	☐	A description of any assumptions or corrections, such as tests of normality and adjustment for multiple comparisons
☐	☒	A full description of the statistical parameters including central tendency (e.g. means) or other basic estimates (e.g. regression coefficient) AND variation (e.g. standard deviation) or associated estimates of uncertainty (e.g. confidence intervals)
☐	☒	For null hypothesis testing, the test statistic (e.g. *F*, *t*, *r*) with confidence intervals, effect sizes, degrees of freedom and *P* value noted<br>*Give P values as exact values whenever suitable.*
☒	☐	For Bayesian analysis, information on the choice of priors and Markov chain Monte Carlo settings
☐	☒	For hierarchical and complex designs, identification of the appropriate level for tests and full reporting of outcomes
☐	☒	Estimates of effect sizes (e.g. Cohen's *d*, Pearson's *r*), indicating how they were calculated

Our web collection on statistics for biologists contains articles on many of the points above.

Software and code

Policy information about availability of computer code

Data collection	For western blotting, ChemiDoc MP System (Bio-Rad), ChemiDoc Touch System (Bio-Rad), and Tanon 4600 (Tanon) were used for protein detection. Immunofluorescence staining images were examined using Leica TCS SP8 confocal microscope (Leica). qRT-PCR data were acquired using LightCycler480 II Real-Time Fluorescence Quantitative PCR system (Roche) and Applied Biosystems 7500 Real-Time Fluorescence Quantitative PCR System (Thermo Fisher Scientific). H&E and IHC staining images were examined using DMi8 microscope (Leica), BX53 microscope (Olympus), Pannoramic MIDI (3DHISTECH). Cell morphology images were examined using IX71 microscope (Olympus). For luciferase assay and ELISA assay, EnVision multimode plate reader (PerkinElmer), TriStar LB 941 (INTERCHIM), and SYNERGY 2 (BIOTEK) were used for data collection. The bioluminescence images were captured using an In Vivo MS FX pro Imaging System (Bruker) and IVIS Lumina imaging station (Perkin Elmer). For biolayer interferometry assay, Octet Red 96 (ForteBio) was used for data collection.
Data analysis	Image Lab software Version 6.0 (Bio-Rad, CA, USA), ImageJ software Version 1.52 (National Institutes of Health, MD, USA), GraphPad Prism software Version 9.0 (GraphPad, CA, USA), Illustrator for BioSequence Version 2.0 (https://ibs.renlab.org/#/home), PyMOL Version 2.2 (The PyMOL Molecular Graphics System), I-TASSER (https://zhanggroup.org/I-TASSER/), CoDockPP Version 2020 (http://codockpp.schanglab.org.cn/), TBtools Version 1.108 (https://github.com/CJ-Chen/TBtools).

For manuscripts utilizing custom algorithms or software that are central to the research but not yet described in published literature, software must be made available to editors and reviewers. We strongly encourage code deposition in a community repository (e.g. GitHub). See the Nature Portfolio guidelines for submitting code & software for further information.

Data

Policy information about availability of data

All manuscripts must include a data availability statement. This statement should provide the following information, where applicable:

- Accession codes, unique identifiers, or web links for publicly available datasets
- A description of any restrictions on data availability
- For clinical datasets or third party data, please ensure that the statement adheres to our policy

The mass spectrometry proteomics data are available via ProteomeXchange (http://proteomecentral.proteomexchange.org/cgi/GetDataset?ID=PXD037460). The 3D structure of the human p85α RHO domain is extracted from the Protein Data Bank archive (https://www.rcsb.org/structure/1PBW). All data supporting the findings of this study are included in the manuscript and its supplementary files are available. Source data are provided with this paper.

Research involving human participants, their data, or biological material

Policy information about studies with human participants or human data. See also policy information about sex, gender (identity/presentation), and sexual orientation and race, ethnicity and racism.

Reporting on sex and gender	The report is available in Supplementary Table 1.
Reporting on race, ethnicity, or other socially relevant groupings	Not applicable.
Population characteristics	The report is available in Supplementary Table 1.
Recruitment	Human glioblastoma specimens were procured from Third Affiliated Hospital of Soochow University. The patients were recruited randomly and underwent tumor removal surgery conducted by Department of Neurosurgery in Third Affiliated Hospital of Soochow University. Stringent measures were taken to ensure the absence of self-selection bias or any other biases in the sample selection process. These cases were selected based on a clear pathological diagnosis as glioblastoma, and were determined to be PTEN wild type by PCR single-strand conformation polymorphism (PCR-SSCP).
Ethics oversight	The study was approved by the Ethics Committee of Third Affiliated Hospital of Soochow University (2019 Science No. 003 and 2022 Science No. 159 (M01)) and informed consent was obtained from all human participants. No participant compensation was provided.

Note that full information on the approval of the study protocol must also be provided in the manuscript.

Field-specific reporting

Please select the one below that is the best fit for your research. If you are not sure, read the appropriate sections before making your selection.

☒ Life sciences ☐ Behavioural & social sciences ☐ Ecological, evolutionary & environmental sciences

For a reference copy of the document with all sections, see nature.com/documents/nr-reporting-summary-flat.pdf

Life sciences study design

All studies must disclose on these points even when the disclosure is negative.

Sample size	The sample size for each experiment is indicated in the legend. Statistical methods were not employed to predetermine the sample sizes. The sample size was chosen empirically based on previous published literature to ensure adequate statistical power for detecting the indicated biological effects.
Data exclusions	No data were excluded.
Replication	Experiments in the article were robustly reproduced, and detailed replicate information was provided in the figure legends.
Randomization	No statistical methods were used for randomization. In mouse experiments exploring the role of target molecules in tumor development, mice were randomly assigned to either the experimental or control groups. In mouse experiments assessing the efficacy of peptide antitumor treatment, mice with comparable tumor sizes and weights were randomly assigned to the experimental group prior to the initiation of treatment. For the remaining studies, experiments were performed in cell lines, and randomization was therefore not appropriate.
Blinding	Investigators were blinded for histological specimen analysis. For experiments without subjective estimation, investigators were unblinded as there was no potential for bias. In other experiments conducted in parallel treatments, investigators were also unblinded due to the absence of interpretation bias.

Reporting for specific materials, systems and methods

We require information from authors about some types of materials, experimental systems and methods used in many studies. Here, indicate whether each material, system or method listed is relevant to your study. If you are not sure if a list item applies to your research, read the appropriate section before selecting a response.

Materials & experimental systems

n/a	Involved in the study
☐	☒ Antibodies
☐	☒ Eukaryotic cell lines
☒	☐ Palaeontology and archaeology
☐	☒ Animals and other organisms
☐	☒ Clinical data
☒	☐ Dual use research of concern
☒	☐ Plants

Methods

n/a	Involved in the study
☒	☐ ChIP-seq
☒	☐ Flow cytometry
☒	☐ MRI-based neuroimaging

Antibodies

Antibodies used	anti-Flag (Sigma Aldrich, #A8592, 1:2000), anti-HA (Roche Applied Science, #3F10, 1:2000), anti-Myc (Santa Cruz Biotechnology, #sc-40, 1:1000), anti-GFP (Santa Cruz Biotechnology, #sc-9996, 1:1000), Streptavidin-HRP (Thermo Fisher Scientific, #434323, 1:1000), anti-NLRP6 (OriGene, #TA337214, 1:1000), anti-PTEN (Santa Cruz Biotechnology, #sc-7974, 1:1000), anti-p85α for immunohistochemistry (Santa Cruz Biotechnology, #sc-376112, 1:100), anti-p85α (Cell Signalling, #13666) for immunofluorescence (1:200), western blot (1:1000) and coimmunoprecipitation (1:1000), anti-pS473-AKT (Cell Signalling, #4060S, 1:2000), anti-pT308-AKT (Cell Signalling, #9275S, 1:1000), anti-AKT (Cell Signalling, #4691S, 1:1000), anti-RBX1 (Santa Cruz Biotechnology, #sc-393640, 1:200), anti-SKP1 (Santa Cruz Biotechnology, #sc-5281, 1:200), anti-OPTN (Novus Biologicals, #NBP1-84682, 1:1000), anti-ATG5 (Cell Signalling, #12994S, 1:1000), anti-LC3 (Cell Signalling, #3868S, 1:1000), anti-Ubiquitin (Cell Signalling, #3936S, 1:1000), anti-Cullin-1 (Abcam, #ab75817, 1:1000), anti-His (Cell Signalling, #9991S, 1:1000), anti-GST (Beyotime Biotechnology, #AG768, 1:1000), anti-β-actin (Sigma Aldrich, #A2228, 1:3000; Cell Signalling, #4970, 1:3000), anti-Rabbit IgG (Beyotime Biotechnology, #A7016, 1:1000), anti-Mouse IgG (Beyotime Biotechnology, #A7028, 1:1000), anti-rabbit IgG HRP-linked Antibody (Cell Signalling, #7074S, 1:1000), anti-mouse IgG HRP-linked antibody (Santa Cruz Biotechnology, #sc-2005, 1:1000), Goat anti-Rabbit IgG (H+L) Cross-Adsorbed Secondary Antibody Alexa Fluor 568 (Invitrogen, #A-11011, 1:500), Goat anti-Mouse IgG (H+L) Cross-Adsorbed Secondary Antibody Alexa Fluor 633 (Invitrogen, #A-21050, 1:500), Goat anti-Mouse IgG (H+L) Highly Cross-Adsorbed Secondary Antibody, Alexa Fluor 568 (Invitrogen, #A-11031, 1:500), and Goat anti-Rabbit IgG (H+L) Highly Cross-Adsorbed Secondary Antibody Alexa Fluor 488 (Invitrogen, #A-11034, 1:500).
Validation	All antibodies in this study were commercially available and validated by manufacturers. Please refer to the data sheet from the vendor's website for technical info by searching the catalog number provided .

Eukaryotic cell lines

Policy information about cell lines and Sex and Gender in Research

Cell line source(s)	Human HEK293T and U251 cell lines were purchased from National Collection of Authenticated Cell Cultures (Shanghai, China). Human LN229 cell line was purchased from Procell Life Science&Technology Co., Ltd. (Wuhan, China). Human LN18 and HS683 cell lines were purchased from Nanjing BEB Laboratories Co., Ltd. (Nanjing, China).
Authentication	Not authenticated in-house, certificate of analysis from source.
Mycoplasma contamination	These cell lines have been tested for mycoplasma contamination by MycoAler Mycoplasma Detection Kit (R&D Systems, #CUL001B) and the results of detection showed that cultured cells were not contaminated by mycoplasma.
Commonly misidentified lines (See ICLAC register)	No commonly misidentified cell lines were used.

Animals and other research organisms

Policy information about studies involving animals; ARRIVE guidelines recommended for reporting animal research, and Sex and Gender in Research

Laboratory animals	BALB/c nude mice (5-6 weeks old, female) were purchased from Laboratory Animal Center of Soochow University. Animals were maintained and bred in a specific-pathogen free (SPF) environment, adhering to standard conditions of temperature (20-26°C) and humidity (40-70%). They were subjected to a strict 12-hour light cycle, with lights on at 08:00 a.m. and off at 08:00 p.m.
Wild animals	No wild animals were used in the study.
Reporting on sex	The findings are not limited to a specific sex. Sex was not taken into account in the study design.
Field-collected samples	No field collected samples were used in the study.
Ethics oversight	All the animal experiment protocols were approved by Ethics Committee of Soochow University.

Note that full information on the approval of the study protocol must also be provided in the manuscript.

Clinical data

Policy information about clinical studies
All manuscripts should comply with the ICMJEguidelines for publication of clinical research and a completedCONSORT checklist must be included with all submissions.

Clinical trial registration	N/A
Study protocol	For the in-house human tumor samples used in our study, the protocol was approved by the Ethics Committee of Third Affiliated Hospital of Soochow University (2019 Science No. 003 and 2022 Science No. 159 (M01)). All studies were performed in accordance with guidelines and regulations by Third Affiliated Hospital of Soochow University, and in accordance with the Declaration of Helsinki Principles.
Data collection	Tumor tissues were collected from patients who underwent tumor removal surgery at Department of Neurosurgery in Third Affiliated Hospital of Soochow University and were diagnosed with glioblastoma during 2008-2022.
Outcomes	No outcome data was used.
